# The Impact of Traditional Food and Lifestyle Behavior on Epigenetic Burden of Chronic Disease

**DOI:** 10.1002/gch2.201700043

**Published:** 2017-10-27

**Authors:** Mustapha U. Imam, Maznah Ismail

**Affiliations:** ^1^ Precision Nutrition Innovation Institute College of Public Health Zhengzhou University Zhengzhou 450001 China; ^2^ Laboratory of Molecular Biomedicine Institute of Bioscience Universiti Putra Malaysia Serdang Selangor 43400 Malaysia

**Keywords:** cancer, cardiovascular diseases, epigenetics, global burdens, type 2 diabetes

## Abstract

Noncommunicable chronic diseases (NCCDs) are the leading causes of morbidity and mortality globally. The mismatch between present day diets and ancestral genome is suggested to contribute to the NCCDs burden, which is promoted by traditional risk factors like unhealthy diets, physical inactivity, alcohol and tobacco. However, epigenetic evidence now suggests that cumulatively inherited epigenetic modifications may have made humans more prone to the effects of present day lifestyle factors. Perinatal starvation was widespread in the 19th century. This together with more recent events like increasing consumption of western and low fiber diets, smoking, harmful use of alcohol, physical inactivity, and environmental pollutants may have programed the human epigenome for higher NCCDs risk. In this review, on the basis of available epigenetic data it is hypothesized that transgenerational effects of lifestyle factors may be contributing to the current global burden of NCCDs. Thus, there is a need to reconsider prevention strategies so that the subsequent generations will not have to pay for our sins and those of our ancestors.

## 1 Global Burden of Noncommunicable Chronic Diseases (NCCDs)

There are already extensive reviews on the global burden of NCCDs in the public domain.^1^, ^2^, ^3^, ^4^, ^5^, ^6^ The consensus that can be surmised from the available data on NCCDs is that their overall prevalence appears to be rising globally and the projections indicate an upward trend. Epidemiological transition data suggests that the morbidity and mortality rate from these diseases is far more than that of communicable diseases in the developed world^7^ and a similar trend is now occurring in the low to medium income countries (LMICs).^8^ Among the NCCDs, cardiovascular diseases (CVDs) are the leading causes of morbidity and mortality, followed closely by metabolic diseases like type 2 diabetes and cancers.^2^, ^9^ By 2030, NCCDs are expected to account for over 70% of all global deaths, out of which 80% will be in the LMICs.^2^, ^10^ Several attempts at understanding the genetics of many NCCDs have yielded better insights into the genetic perturbations that are associated with these disease, albeit the outcomes have been disappointing; the evidence from genome wide association studies, family linkage analyses, and twin cohort studies suggests that genetic abnormalities only account for a very small risk of NCCDs.^11^, ^12^ With these insights, lifestyle factors have received closer scrutiny and many NCCDs have now come to be associated with specific lifestyle choices especially diet.^2^, ^3^, ^5^, ^13^, ^14^

The World Health Organization (WHO) advises that to achieve overall improved health around the world, national health systems must balance caloric intake with physical activity levels to attain an optimal health. This balance, they argue, can be promoted by avoiding unhealthy diets, tobacco use, and harmful use of alcohol, while increasing physical activity levels.^2^, ^3^ The American Heart Association also recommends the use of diets rich in vegetables, fruits, fish, and whole‐grain, high‐fiber foods, while limiting the intake of saturated fats to 7% of energy, trans fats to 1% of energy, and cholesterol to 300 mg d^−1^ to curb the burden of cardiovascular diseases (CVDs), which are the leading causes of morbidity and mortality among the NCCDs.^13^ They also recommend moderate alcohol intake, avoidance of smoking, and reduction in the intake of foods/beverages with added sugar, and the consumption of fat‐free or low‐fat (1% fat) dairy products and foods with little or no salt.^2^ The necessity of salt consumption is however underlined by the fact that it is an important means of preventing iodine deficiency,^15^ and thus must be balanced. These recommendations are in line with those of WHO, suggesting a convergence in thinking on how to properly manage NCCDs. Additionally, Heidemann et al.^16^ had demonstrated that adoption of a western lifestyle characterized by consumption of energy‐dense foods that are rich in red meat, processed meat, refined grains, and sweets was associated with higher risk of mortality from CVD and all‐cause mortality, compared with a more traditional diet characterized by consumption of vegetables, fruit, legumes, fish, poultry, and whole grains. The risk of type 2 diabetes mellitus was also shown to be substantially higher in individuals who consume such western diets.^11^, ^17^

CVD accounts for more than 50% of all cases and death due to NCCDs globally including in LMICs.^18^ This was not always the case and CVD accounted for less than 10% of morbidity and mortality even up to the 19th century.^6^, ^18^ In the 20th and 21st centuries, the burden of CVD saw an upward trend that is expected to continue in this direction despite advances in healthcare systems around the world.^19^ Lifestyle factors, including unhealthy diet and physical inactivity,^6^, ^16^, ^20^, ^21^, ^22^ which were not prevalent during the 18th and 19th centuries have been blamed for this rising burden of CVD around the world. Extensive studies have also shown that lifestyle interventions may significant alter the progression of CVD and several recommendations have been given toward such goals.^3^, ^13^, ^23^, ^24^, ^25^, ^26^, ^27^ Despite these recommendations, the declining CVD mortality at least in the developed world in recent years may not be down to adoption of these recommendations and reduced disease burden, but largely due to improved quality of care and treatment.^28^, ^29^ In LMICs, however, morbidity and mortality from CVD still remain high, a trend that is blamed on traditional CVD risk factors^6^, ^18^ although a growing body of epigenetic data suggests inherited influences may likely be contributing to the overall burden of CVD and other NCCDs.^30^ Periconceptional and intrauterine conditions can induce molecular reprograming events in the form of DNA methylation changes and/or histone modifications to optimize survival of the fetus, only for these changes to predispose to adult NCCDs postnatally.^31^

Furthermore, type 2 diabetes mellitus is on the increase globally. In 2015, there were 415 million adults living with diabetes, and the number is expected to rise to 642 million by 2040.^32^ Type 2 diabetes in the pediatric population is increasingly becoming prevalent largely driven by obesity, and accounts for a significant number of diabetics in the developed countries.^33^ This growing burden has been the trend for a long time now and has not shown any signs of slowing down.^1^, ^34^ Interestingly, the increase in type 2 diabetes incidents is expected to be highest in the LMICs, where such metabolic disease was relatively low before the 1980s.^33^ Despite the dismal statistics, a huge number of diabetics are believed to be undiagnosed especially in Africa and Middle East, where almost two‐thirds of all diabetics do not know they have the disease. In fact, the International Diabetes Federation estimates that there are as much undiagnosed diabetics as those that are diagnosed. These undiagnosed cases are more prone to developing complications, thus adding to the economic burden of the disease through direct medical costs and lost productivity.^32^, ^35^ Just like CVD burden, that of type 2 diabetes is also closely linked with unhealthy lifestyle choices like unhealthy diets and physical inactivity.^17^, ^36^, ^37^ Additionally, the global burden of cancers has been growing exponentially, with 2012 estimates indicating that more than 14 million cancer cases occurred in that year with additional 8 million cancer deaths, majority of which occur in the LMICs.^38^, ^39^ Breast, colon, and cervical cancers are the commonest among females, while lung, prostate, and colon cancers are the commonest among males.^4^ Cancers too have been associated with unhealthy lifestyles.^40^

Overall, NCCDs especially CVDs, type 2 diabetes and cancers are taking a heavy toll on the health of many individuals especially in the LMICs. Sadly, the economic impact of these diseases is helping to impoverish these countries whose economies are already bad. Since the initial observations of the rising trend in global NCCDs burden, advances in healthcare systems around the world and lifestyle recommendations have not been met with a corresponding reduction in the burden of overall NCCDs. This is despite passionate advocacy that a large burden of these NCCDs could be reduced with adherence to set recommendations of dietary and lifestyle modifications.^2^, ^3^, ^13^, ^23^, ^24^, ^26^, ^35^, ^36^, ^37^, ^38^, ^41^, ^42^ However, as we will argue later in this review, ancestrally inherited and/or intrauterine acquired epigenetic influences may be complicating the burden of NCCDs among different populations, who have either inherited and/or undergone some experiences known to confer transgenerational increase in risk of diseases. Even the International Diabetes Federation appears to have acknowledged in recent years that policy shifts to acknowledge the contributions of intrauterine and early life epigenetic influences may greatly influence the control of type 2 diabetes.^43^ Thus, it is our strong conviction that nonconsideration of the contributions of epigenetic influences on the burden of NCCDs may be the reason why lifestyle recommendations have so far not yielded the desired outcomes in terms of reduced burden of these diseases.

## 2 Changing Ancestral Life Style Factors and Implications on Disease Burden

Dietary and other lifestyle factors have significantly changed over the past few centuries to be what we have today. The sources and preparation of food for human consumption generally parallel the technologies available to humans at any given time in history. Thus, it was common practice to engage in manual farming activities and to travel over long distances on foot in search of food and other livelihood from the Paleolithic periods up to the preindustrial period. There are indications that the lifestyles of preindustrial humans that lived just prior to and around the 18th century closely resembled those of the late Paleolithic hunter‐gatherer humans than those of present day humans (**Table** **1**).^28^, ^44^, ^45^, ^46^, ^47^, ^48^ Moreover, evidence from Australian Aborigines, who lived as hunter‐gatherers into the 20th century had provided insights into the lifestyles of past hunter‐gatherers.^46^

**Table 1:** Differences in physical activity dietary consumption between preindustrial and present day humansa)

	Preindustrial humans	Present day humans
Physical activity	High	Low
Protein	High	Low
Carbohydrate	Similar	
Refined sugars	Low	High
Glycemic load	Low	High
Fat	Low	High
Saturated fats	Low	High
Trans fats	Low	High
Monounsaturated fats	High	Low
Polyunsaturated fats	High	Low
Harmful alcohol use	Low	High
Cholesterol	High	Low
Fiber	High	Low
Sodium/salt	Low	High
Calcium	High	Low
Ascorbic acid	High	Low
Plant based foods including fruits and vegetable	High	Low
Grains	Low	High

^a)^ Source: Refs. 44, 45, 47, 49.

Similarly, preindustrial humans were far more physically active and consumed foods that were more natural than what humans consume now.^45^, ^48^, ^49^ Anthropological analyses using muscular insertion sites, the area of articular surfaces, and the cortical thickness and cross‐sectional shape of long bone shafts of paleolithic and preindustrial humans have demonstrated that they were stronger and more active than their present day descendants. The overall physical activity levels and healthy nutritional status of humans through the paleolithic to the preindustrial periods may have resulted in very little adverse epigenetic influences that would have influenced chronic disease risk in successive generations during these periods. Such healthy lifestyles and physical activity levels would have undoubtedly induced beneficial epigenetic influences leading to reduced disease risk in subsequent generations.^50^, ^51^ However, longevity is notably higher among present day humans compared to the preindustrial humans despite the seemingly healthier lifestyles of those people possibly because the major causes of deaths were acute factors;^52^ there were no antibiotics against infectious diseases, and modern medical equipment needed to take care of injured persons as a result of war, violence or accidental trauma were not available. Moreover, seasonal epidemics may have caused significant deaths among the preindustrial humans.^53^ These factors coupled with recent advances in overall healthcare delivery would have put present day humans at an advantage in terms of longevity over the preindustrial humans.

Humans have undergone several developmental phases that correspond with important epidemiological transitions over the last three centuries when public health information began to be recorded aggressively in attempts to improve wellbeing and health. Thus, in accordance with the phases of epidemiologic transition, namely pestilence and famine, receding pandemics, degenerative and man‐made diseases, and the phase of delayed degenerative diseases,^6^, ^18^ epigenetic influences may have been carried over across several generations, with possible implications on adult disease outcomes.^31^

On average, the body‐mass index (BMI) of people is higher in recent years than it used to be in the past.^54^ However, we cannot rule out contributions from our ancestors. The fact that some ethnic populations do not lose the risks of certain diseases when they move to other countries further supports this hypothesis. Asians who have moved to the US have been shown to have similar CVD risks to other Asians living in Asia than other ethnic groups living in the US. In fact, they have been shown to be prone to developing NCCDs like CVDs with minimal western environmental influences.^55^ Similarly, Australian Aborigines are easily prone to developing obesity, type 2 diabetes and CVD when they transition to western lifestyles.^46^ Genetic risk can only account for a small proportion of these risks due to lack of overwhelming evidence linking cases of CVDs to genetic predisposition among the Asians or Australian Aborigines. In some cases, however, critical changes during development have been shown to modulate disease risk irrespective of genetics. For example, the risk of multiple sclerosis is higher in the Northern than the Southern hemisphere, and the original risk of developing such remains when an individual migrates after puberty. On the other hand, migration during childhood confers similar risk as the host community.^56^ It is thought that protective factors in the Southern hemisphere like maternal nutritional status, exposure to sun, helminths, and other parasites are able to modify risk only during critical developmental windows, in support of extragenetic programing of disease risk.^57^ Besides, there is evidence that childhood environmental factors can program the risk of adult NCCDs, which can be transmitted across multiple generations.^58^, ^59^, ^60^

## 3 How Nutritional Epigenetics Underlies the Growing Burden of Chronic Disease

The world is experiencing ever increasing NCCDs burden despite extensive documentation of traditional risk factors known to promote these diseases and attempts at implementing counter‐measures in many countries around the world. We hypothesize that in view of the growing epigenetic evidence (detailed below) linking many of the environmental experiences of our ancestors in the last three centuries to adult NCCDs, the current global burden of diseases may have been shaped by inherited experienced that may have been compounded by our unhealthy lifestyle choices.^18^ Moreover, at no time in human history have we experienced or are projected to experience such a huge NCCDs burden that is attempting to defy all known preventive measures. Thus, cumulatively inherited epigenetic modifications over time could have affected our risk of diseases and its overall burden, as explained below:

### 3.1 Starvation

Starvation around the time of conception and early gestation in humans has epigenetic implications (**Table** **2**), including the hypomethylation of the lymphocyte IGF2 gene^61^, ^62^ and hypermethylation of the lymphocyte IL10, LEP, ABCA1, GNASAS, and MEG3 genes in adult life.^61^ Human exposure to perinatal famine can also produce distinct DNA methylation patterns related to genes involved in eye development (CDH23 and RFTN1), forebrain formation (SMAD7), growth (INSR) and sustaining early pregnancy (KLF13). Many more epigenetic alterations have been documented due to perinatal famine exposure in humans, with consequent increases in offspring's' risk of diseases during childhood and even adulthood.^59^, ^60^, ^63^, ^64^, ^65^, ^66^, ^67^, ^68^, ^69^, ^70^, ^71^, ^72^, ^73^, ^74^, ^75^, ^76^, ^77^, ^78^, ^79^, ^80^, ^81^, ^82^, ^83^, ^84^, ^85^ Data from animal experimentation have also provided more insights into how nutritional deficiency can induce epigenetic alterations (Table 2).^86^, ^87^, ^88^, ^89^, ^90^, ^91^, ^92^, ^93^, ^94^, ^95^ For example, protein restriction in pregnant rodents was shown to produce hypomethylation of the hepatic glucocorticoid receptor (GR) and peroxisome proliferator‐activated receptor alpha (PPARα) gene promoters, with consequently increased expression of these genes,^87^ even across several generations^88^, ^90^ through reprograming of the germline methylation levels.^89^ The placenta plays a central role in nutrient transfer between mother and fetus during intrauterine life and helps to maintain pregnancy through other mechanisms, and could be a target of the epigenetic modifications due to starvation mediated via changes to methylation levels of imprinted genes and microRNAs associated with genes involved in a wide range of activities from placental nutrient transfer and fetal development to multiple diseases.^92^ Similarly, perinatal deficiency of methyl donors like folic acid and B vitamins, and other nutrients could influence methylation levels in offsprings and their risk of disease in adult life (**Table** **3**).^93^, ^96^, ^97^, ^98^, ^99^, ^100^, ^101^, ^102^, ^103^, ^104^, ^105^, ^106^, ^107^, ^108^, ^109^, ^110^, ^111^, ^112^, ^113^, ^114^, ^115^, ^116^, ^117^, ^118^, ^119^, ^120^, ^121^, ^122^, ^123^, ^124^, ^125^, ^126^, ^127^, ^128^, ^129^, ^130^, ^131^, ^132^, ^133^, ^134^, ^135^, ^136^, ^137^, ^138^, ^139^ Perinatal deficiency of iron has been associated with the increased risk of high fat diet induced insulin resistance in offsprings.^99^, ^100^ Such deficiencies of protein, methyl donors, and other essential nutrients, which would have been common during periods of starvation and may have been the basis for the epigenetic sequelae widely associated with the Dutch hunger, Norbotten, and other cases of famine (Table 2). Despite the growing body of evidence showing epigenetic traits and phenotypic changes induced by perinatal exposure to famine, these epigenetic traits have not been linked to NCCDs development on a public health scale possibly because of our limited understanding of their roles in causing disease in the past.

**Table 2:** Epigenetic alterations induced by lifestyle factors that may have shaped human disease history

Lifestyle factor	Species/study type	Experimental setting [reference]	Epigenetic effects and alterationsa)
Starvation	Human/cohort study	Perinatal and early postnatal exposure to Dutch winter hunger (1944–1945)^58^, ^60^, ^62^, ^63^, ^64^, ^65^, ^69^, ^70^, ^71^, ^77^, ^141^	Increased offsprings' risk of obesity, diabetes, hypertension, CVD, chronic lung disease, cancers, death, and cognitive and mental disorders in offsprings six decades later
			Higher risk of insulin resistance and CVD when offsprings are bottle fed instead of breast feeding
			Hypomethylation of lymphocyte IGF2 DMR
			Changes in methylation levels of 181 DMRs of genes involved in growth and metabolism including SMAD7, CDH23, INSR, RFTN1, CPT1A, and KLF13
	Human/observational case control study	Association of lower weight at birth and higher weight at 18 years with the risk of metabolic syndrome in the general Swedish population at 58 years^79^	Low birth weight and higher catch up growth at 18 years was associated with higher BMI, blood pressure insulin resistance, LDL and triglycerides, and lower HDL at 58 years
	Human/cohort study	Association between body size at birth and intake of foods and macronutrients in adulthood in Helsinki birth cohort^80^	Lower birth weights were associated with lower intake of fruits and berries, carbohydrates, sucrose, fructose, and Fibre, and higher intake of fat in adulthood (56–70 years)
	Human/cohort study	Risk of insulin resistance and CVD in Indian children with history of low birth weight and obesity at 8 years^78^	Maternal undernutrition resulted in low birth weight, which was associated with increased adiposity at 8 years
			Higher risk of insulin resistance and CVD in children who were born small but grew heavy (or tall) afterward
	Human/cohort study	Association between change in parents' and grandparents' early food supply and cardiovascular or diabetes mortality of the grandchildren, among three cohorts born in 1890, 1905 and 1920 in Överkalix parish in northern Sweden up until death or 1995^74^, ^76^, ^82^	Decreased cardiovascular mortality with decreasing availability of food for fathers and paternal grandmothers during slow growth period (9–12 years)
			Decreased risk of diabetes with exposure to famine in paternal grandfathers during slow growth period
			Increased diabetes mortality and overall mortality with increased food availability for paternal grandfather during slow growth period
			Increased risk of cardiovascular mortality among females whose paternal grandmothers had sharp change in food supply during their slow growth period from one year to next
	Human/cohort study	Association between mothers' food availability during pregnancy and mortality at 40–70 years among those born between 1805 and 1849 in Skellefteå parish, Sweden^75^	Increased risk of death with famine exposure or overabundance in mothers during the early stages of pregnancy, and food abundance or famine exposure toward the end, respectively
	Human/cohort study	Association between maternal undernutrition and risk of adult disease in low and middle income countries (Brazil, Guatemala, India, the Philippines, and South Africa)^59^	Increased risk of shorter adult height, less schooling, reduced economic productivity, and lower offspring birth weight
			Higher birth and childhood weights were associated with adult body‐mass index and blood pressure
			Lower birth weight and undernutrition in childhood were associated with high glucose concentrations, blood pressure, and harmful lipid profiles after adjusting for adult BMI and height
			Higher birth weight was associated with poorer lung function and with the incidence of some cancers (breast, prostate, hemopoietic, and colorectal cancers)
			Childhood undernutrition was associated with mental illness, and lower height‐for‐age at 2 years was associated with lower human capital
	Human/cohort study	Association between prenatal undernutrition (including reduced methyl donor availability) and adult mortality in children born in the hungry seasons (June–October) between 1949 and 1994 in the Gambia^81^, ^83^	Increased methylation levels of metastable epialleles (BOLA3, LOC654433, EXD3, ZFYVE28, RBM46, and ZNF678) in offspring's lymphocytes and hair follicles
			Increased risk of premature death among 15–35 year old offsprings
	Human/cross‐sectional study	Association between prenatal malnutrition among Chinese born in 1954–1964 and adult Chronic disease in adolescents and adults^67^, ^68^, ^72^, ^84^, ^85^	Shorter adult heights and less economic prosperity
			Increased risk of metabolic syndrome, schizophrenia and death in both males and females, and increased risk of mental illness in females
			Metabolic risk higher in nutritionally rich environment
	Human	Association between prenatal famine and type 2 diabetes at 40 years of age in those exposed the Ukrainian 1930–1938 famine^73^	Increased risk of type 2 diabetes in both men and women
	Human/cohort study	Associations between prenatal exposure to famine during the Nigerian civil war (1967–1970) and hypertension, glucose intolerance, and overweight 40 years after^66^	Increased risk of adult hypertension, insulin resistance, and obesity
	Rat	Dietary protein restriction during pregnancy^86^, ^87^, ^88^, ^89^, ^93^	Increased preference for fatty food than high‐carbohydrate food in male and female rat offsprings at 12 and 30 weeks
			Higher plasma insulin in males at 12 weeks
			More gonadal fat in male offsprings at 30 weeks
			Decreased hypothalamic expression of galanin‐2 receptor in female offspring at 12 weeks
			Decreased hepatic expression (mRNA and protein) of sterol response element binding protein (SREBP‐1c), with blunted response after high fat feeding at 12 weeks
			Increased Igf2 and H19 gene expression in the liver of day 0 male offspring
			Hypermethylation of IGF2/H19 DMRs at birth
			Increased expression of Dnmt1 and Dnmt3a, and Mbd2 at birth
			Hypomethylation of the hepatic glucocorticoid receptor (GR) and peroxisome proliferator‐activated receptor alpha (PPARa) gene promoters and increased expression of these genes, and those of acyl‐CoA oxidase and phosphoenolpyruvate carboxykinase in F1 and F2 offsprings
	Mice	Dietary protein restriction during pregnancy^90^, ^94^, ^95^	Hypercholesterolemia, hyper‐triacylglycerolemia, hyperglycemia, glucose intolerance, hyperinsulinemia and insulin resistance, increased leptin and resistin, increased adiposity, and leptin resistance characterized by altered expression of neuropeptide Y and proopiomelanocortin (POMC) in F1 and F2 offsprings
			Lower body weight/adiposity and higher food intake
			Lower levels of leptin promoter DNA methylation, and fasting mRNA and protein levels, but more pronounced induction postprandially
			Hypermethylation of the hepatic liver X‐receptor alpha promoter with reduced mRNA level of Lxra and its target genes Abcg5/Abcg8
	Mice	Dietary restriction (50% less diet) during pregnancy^91^, ^92^	Locus‐specific hypomethylation of sperm DNA at intergenic nonrepetitive regions and CpG islands in F1 adult males, which is lost in F2 offsprings, although locus‐specific gene expression is still altered in tissues including brain and liver
			Increased expression of hepatic lipid oxidation genes including PPARα, Pgc1α and Pgc1β, and downregulation of genes involved in lipid synthesis including Scd1, Srebp1, and Dgat1
			Widespread changes in placental miRNAs and DNA methylation patterns of genes associated with immunological, metabolic, gastrointestinal, cardiovascular, and neurological chronic diseases, as well as those involved in transplacental nutrient transfer and fetal development
High fat diet/obesity	Human/cohort study	Parental high obesity^167^, ^168^, ^169^	Paternal obesity associated with decreased methylation levels at the IGF2, MEST, PEG3, and NNAT DMRs in cord blood
			Paternal obesity associated with decreased methylation levels at the MEG3, NDN, SNRPN, and SGCE/PEG10 DMRs, and higher methylation levels of MEG3‐IG and H19 DMRs in sperm
			Maternal obesity associated with increased methylation of PLAGL1 DMR and decreased MEG3 DMR in cord blood
	Human/cohort study	Childhood obesity^206^, ^207^, ^208^, ^209^, ^210^, ^211^	Increased DNA methylation of the CASP10, CDKN1C, EPHA1, HLADOB3, IRF5, MMP9, MPL, NID1, retinoid X receptor‐α, IGF2/H19, and POMC, and lower methylation of LINE1 in children are strongly associated with childhood obesity
			Hypo‐ and hypermethylation and gene expression changes of the TACSTD2 gene at birth associated with childhood obesity
	Rat	Maternal high fat diet prior to, and during pregnancy and lactation^127^, ^131^, ^172^, ^176^, ^178^, ^179^, ^182^, ^183^, ^184^, ^185^, ^187^, ^189^, ^194^, ^199^	Higher placental weight, birth weight, and blood glucose at birth
			Increased weight gain, insulin resistance, lipid profiles, GLP‐1, serum leptin, and fat preference
			Inhibited osteogenesis and decreased expression of bone homeodomain‐containing factor A10 (HoxA10), osteocalcin, alkaline phosphatase, Runx, and osterix and increased expression of fatty acid binding protein and PPARγ
			Hypermethylation of bone HoxA10 promoter
			Higher phosphorylated 4EBP1 (T37/46 and S65) and rpS6 (S235/236) in the placenta
			Lower phosphorylation of AMPK and eIF2alpha in the placenta
			Hypomethylation of PPARγ, FAS, adiponectin and leptin gene promoter in white adipose, with consequently increased expression (mRNA) of PPARγ, FAS, and adiponectin, and decreased leptin expression
			Decreased hepatic expression of Wnt1 (mRNA) and nuclear β‐catenin (protein)
			Decreased hepatic mRNA expression of circadian (CLOCK, BMAL1, REV‐ERBα, CRY, PER) and metabolic (PPARα, SIRT1) genes
			Differential expression of H3K4me3 and H3K27me3 in hepatic PPARα promoter
			Decreased hepatic Wnt1 gene promoter H4 and H3 acetylation and increased H3K9 methylation
			Increased hepatic expression (mRNA) of gluconeogenic genes (PEPCK1, G8Pase, Cebpα, Cebpβ, Srebp‐1a, and Pgc1a)
			Decreased H3Ac, H3K4me2, H3K9me3, and H3K27me3 in hepatic PEPCK1 promoter
			Increased H4Ac and H3K4me2 in hepatic PEPCK1 coding and upstream regions, and reduced H3K9me3 in hepatic PEPCK1 coding region
			Increased hepatic TBARs, and expression (mRNA) of p16INK4a and Cox2
			Increased expression (mRNA) of hepatic lipogenesis, oxidative stress, and inflammatory genes
			Decreased hepatic expression (mRNA) of Pon1, Pon2, Pon3, and Sod1, Gpx1
			Increased H4Ac and H3K4me2 in hepatic Pon1 promoter
			Increased hepatic mRNA levels of peroxisome proliferator activated receptor‐alpha, carnitine palmitoyl transferase‐1a and Igf2
			Decreased expression of ≈23 hepatic miRNA levels (≈1.5–4.9‐fold) including miR‐709, miR‐122, miR‐192, miR‐194, miR‐26a, let‐7a, let‐7b, and let‐7c, miR‐494 and miR‐483
			Increased expression (mRNA) of hepatic Cdkn1a *and hypomethylation of its* promoter
			Increased expression (mRNA) of DAT and MOR in the brain
			Decreased expression (mRNA) of hypothalamic NPY, proopiomelanocortin, leptin receptor and STAT3
			Global hypomethylation in prefrontal cortex
			Increased expression of orexigenic peptides, galanin, enkephalin, and dynorphin, in the paraventricular nucleus and orexin and melanin‐concentrating hormone in the perifornical lateral hypothalamus
			Decreased expression (mRNA) of Pgc‐1α and its target genes (Glut4, Cox4, and Cyt c) in skeletal muscle
			Hypermethylation of the skeletal muscle Pgc‐1α and hypothalamic POMC promoters
	Rat	Maternal low protein diet prior to, and during pregnancy and lactation, and postnatal high fat diet^177^	Increased adipose growth rate, insulin resistance and IGF2 mRNA and methylation
	mice	Paternal high fat diet^174^, ^188^	Impaired glucose tolerance
			Altered expression of 642 pancreatic islet genes involved in calcium‐, MAPK‐ and Wnt‐signaling pathways, apoptosis and the cell cycle
			Hypomethylation of the pancreatic *Il13ra2* gene
			Global hypomethylation of germ cell DNA in F0 mice
			Altered testes mRNA (414 genes involved in nitric oxide and ROS pathways, Sertoli cell junction signaling, EIF2 signaling, NF‐*κβ* signaling and inflammatory response, lipid metabolism, and carbohydrate metabolism), and sperm and testes microRNA (11 microRNAs mostly involved in metabolic disease, cell death, production of ROS, DNA replication, NF‐κB signaling, p53 signaling, recombination and repair, lipid metabolism, spermatogenesis, and embryonic development) expression
	Mice	High fat diet during pregnancy and lactation^143^, ^170^, ^173^, ^175^, ^180^, ^186^, ^192^, ^195^, ^196^, ^198^, ^200^	Increased body weight, body fat content, inflammatory markers and serum insulin and leptin concentrations
			Decreased adiponectin expression and increased leptin in white adipose tissue
			Lower H3K9Ac and higher H3K9me2 in adiponectin promoter of white adipose tissue
			Increased CD‐68, chemokine receptor‐2 and TNFa mRNA, and decreased GLUT‐4 mRNA in subcutaneous adipose tissue
			Higher H4K20me at leptin promoter of white adipose tissue
			Increased hepatic expression (mRNA) of PEPCK, PGC1α, JNK, and Ikbkb
			Increased hepatic H3K14ac and H3K9me3
			Decreased hepatic global histone methylation and H3K9Me2 in F2 mice
			Decreased expression of hepatic miR‐122 and increased expression of miR‐370
			Decreased Histone methylation in promoters of hepatic LXRa and ERO1‐a in F2 mice
			Upregulation of hypothalamic toll‐like receptor (Tlr) 4 signaling cascade including c‐Jun N‐terminal kinase 1 and IκB kinase‐β inflammatory pathways
			Increased expression (mRNA) of DAT in the ventral tegmental area, nucleus accumbens, and prefrontal cortex and in the hypothalamus
			Increased expression (mRNA) of MOR and PENK in nucleus accumbens, prefrontal cortex, and hypothalamus
			Global and gene‐specific (DAT, MOR, and PENK) promoter DNA hypomethylation in the brain
			Increased H3K9me and decreased H3Ac in the MOR promoter region of the brain
			Global DNA hypomethylation in female placenta
			Differential methylation of placental Igf2r regions that serve as binding sites for important transcription factors including Pax4, Smarca3, Vbp, Pax6, Yy1, Oct1, Nrf2/Arp, Ppar/Rxr, Egr3, Rxr, Mzf1, Sry/Sox9, Gcm1, Stat6, Nudr/Deaf‐1, and altered expression of placental metabolism genes including Dio3, Rtl1, Dlk1, Slc22a1, Slc22a2, Slc22a3 especially in the female placenta
			Hypermethylation of the leptin promoter in F0 oocytes and female F1 livers, and increased hepatic expression (mRNA)
			Hypomethylation of the Ppar‐α promoter in F0 oocytes and F1 female livers, and decreased hepatic expression (mRNA)
			Hypermethylation level of Ppar‐α promoter in F1 oocyte
	Mice	High fat diet during pregnancy and lactation in successive generations (F0–F2)^181^	Increased body weight in female mice in F1 and F2 generations (most severe in F2)
			Increased macrophage infiltration and inflammatory gene expression in adipose tissue
			Hypomethylation of the adipose tissue promoters of Tlr1, Tlr2, and linker for activation of T cells
	Mice	Embryonic *Cited2* deletion + Maternal high fat diet^197^	Increased penetrance of cited2‐induced defects including cardiac malformation, adrenal agenesis, and other defects
			Decreased expression (mRNA) of Pitx2c in Cited2‐deficient embryos
	Macaque	Maternal high fat diet^190^, ^191^, ^193^	Increased hepatic Npas2 *expression (mRNA)*
			Increased H3K14ac in Npas2 promoter
			Increased hepatic GCN5 (mRNA), global H3K14ac and decreased HDAC III (mRNA) and SIRT1 (mRNA, protein and activity) expression
			Increased expression of downstream hepatic genes modulated by SIRT1 including PPARΑ, PPARG, SREBF1, CYP7A1, FASN, and SCD
			Increased H3K14ac and DBC1‐SIRT1 complex in fetal livers
			Increased hypothalamic POMC mRNA expression, and decreased agouti‐related protein mRNA and peptide levels
			Increased hypothalamic proinflammatory cytokines, including IL‐1β and IL‐1 type 1 receptor
White rice	Rat	Maternal white rice consumption for 8 weeks before and throughout gestation and lactation^226^	Worsened glucose tolerance
			Reduced serum adiponectin levels, and increased weights, homeostatic model assessment of insulin resistance, serum retinol binding protein‐4 levels, and leptin levels
			Altered expression of insulin signaling genes in the liver, muscle, and adipose tissues
Exercise/weight loss	Human/cohort study	Three to six month exercise in men^233^, ^234^	Differential methylation of 7663 genes, including 18 obesity‐related genes
			Global and genome‐wide sperm DNA methylation changes in genes related to diseases like schizophrenia and Parkinson's disease
	Human/cohort study	Differences in methylation levels among children born to the same women before and after weight loss due to bariatric surgery^237^, ^239^, ^240^, ^241^	Lower birth weight, and decreased childhood obesity, insulin resistance, cholesterol, leptin and CRP in offsprings born after weight loss
			Differential methylation and changes in expression of 5698 genes involved in glucoregulatory, inflammatory, and vascular disease pathways in offsprings born after weight loss
			Differential methylation of 23 449 genes (HLA‐DQA1, HLA‐DQB1, and TSPAN18 were most significant), and changes in expression of 3074 genes, involved with insulin receptor signaling, type 2 diabetes signaling, and leptin signaling in obesity in offsprings born after weight loss
	Rat	Maternal high fat diet + exercise during pregnancy and lactation^172^	Attenuation of high fat diet‐induced decreases in expression of Pgc‐1α and its target genes (Glut4, Cox4, and Cyt c) in skeletal muscle
Smoking	Human/cohort study	Long term epigenetic effects of smoking^252^	Long term lymphocyte DNA methylation changes in over 2600 CpG sites annotated to 1405 genes affecting pulmonary function, and risks of cancers, inflammatory diseases and heart disease, which lasted over 30 years; quitting reversed some of these effects within five years
	Human/cohort study	Association between paternal smoking around the slow growth period and epigenetic changes in children in the Avon Longitudinal Study of Parents and Children (ALSPAC) cohort^144^	Higher male offsprings BMIs by age 9
	Human/cohort study	Association between maternal smoking and infant blood methylation^253^	Altered methylation of 185 CpGs of 110 genes, including FRMD4A, ATP9A, GALNT2, and MEG3, implicated in processes related to nicotine dependence, smoking cessation, and placental and embryonic development
	Human/cohort study	Association between maternal smoking and methylation changes in children from kindergarten and first graders^259^	Lower methylation of AluYb8
			Lower LINE1 methylation in children with the GSTM1‐null genotype
			Higher methylation in children with the GSTM1‐present genotype
			Differential methylation of CpG loci in eight genes; two validated genes showed increased methylation
	Human/cohort study	Association between grandmother's, mother's (before or during pregnancy using cotinine measured at 18 weeks gestation), and father's (before conception) smoking history, and methylation at these 26 CpGs mapped to 10 genes (GFl1, AHRR, HLA‐DPB2, MYO1G, ENSG00000225718, CNTNAP2, EXT1, TTC7B, CYP1A1 and RUNX1) in the Norwegian Mother and Child Cohort Study^260^	Differential methylation changes only when mother continued smoking during pregnancy past gestational week 18
	Human/cohort study	Association between maternal smoking and placental and cord blood methylation^103^, ^254^, ^255^, ^256^, ^257^, ^261^	Fetal growth restriction
			Decreased global DNA methylation
			Altered methylation patterns within the P2 promoter of *IGF2*
			Higher methylation at the IGF2 DMR
			Increased CYP1A1 expression
			Hypomethylation of CpG sites on CYP1A1 promoter immediately proximal to the 5′‐xenobiotic response element transcription factor binding element
			Altered expression of 623 genes and the methylation of 1024 CpG dinucleotides
			Differential methylation of 23 CpGs mapped to eight genes: AHRR, GFI1, MYO1G, CYP1A1, NEUROG1, CNTNAP2, FRMD4A, and LRP5
			Downregulation of placental miR‐16, miR‐21, and miR‐146a
	Rat	Association between maternal nicotine exposure and asthma^258^	Increased asthma risk in F2 offsprings
			Decreased lung PPARγ expression (mRNA)
			Increased H3 Acetylation and decreased H4 acetylation in the lung
			Increased DNA methylation, and H3 and H4 acetylation in testis
			Decreased DNA methylation and increased H4 acetylation in the ovary
Alcohol	Human/cohort study	Association between maternal alcohol and DNA methylation in whole blood/buccal tissues of children (1–16 years)^265^, ^267^	Hypomethylation of the KvDMR1 and PEG3 DMR
			Differential methylation of 269 CpGs on multiple genes related to protocadherins, glutamatergic synapses, and hippocampal signaling
	Human/cohort study	Association between periconceptional alcohol use in parents and cord blood methylation levels^266^	Hypomethylation of the DAT gene promoter due to maternal (before and during pregnancy) and paternal (before conception) alcohol use
			Hypomethylation of the SERT gene promoter due to maternal (before and during pregnancy) alcohol use
			Hypermethylation of the MeCP2 promoter due to due to maternal (during pregnancy) alcohol use
	Mice	Maternal alcohol exposure in early gestation^120^, ^272^	Decreased embryonic IGF2 promoter methylation and IGF 2 expression
			Decreased prenatal growth
			Increased mortality, and digit and vertebral malformations
			Increased miR‐467b‐5p in Slc17a6 promoter resulting in hypomethylation of hippocampal H3K4me3 at the Slc17a6 promoter, with corresponding increased mRNA levels of hippocampal Slc17a6, although its protein product VGLUT2 was decreased
	Rat	Maternal ethanol exposure during pregnancy^110^, ^112^, ^114^, ^128^, ^268^, ^269^, ^270^, ^271^	Behavioral, learning and memory deficits, and decreased birth and brain weights, and incisor emergence
			Higher plasma methionine and prolactin concentrations
			Higher pituitary weight, pituitary prolactin protein and mRNA
			Lower pituitary levels of dopamine D2 receptor (D2R) mRNA and protein, and hypermethylation of the pituitary D2R promoter
			Increased pituitary mRNA levels of DNA methylating genes (DNMT1, DNMT3b, MeCP2) and histone modifying genes (HDAC2, HDAC4, G9a)
			Lower whole brain Mtr and Mat2a mRNA, hippocampal Mtr and Cbs mRNA in males
			Higher hippocampal Mtr, Mat2a, Mthfr, and Cbs mRNA in females
			Lower hippocampal Nr3c1 mRNA and NGFI‐A protein in females
			Higher hypothalamic Slc6a4 mean promoter methylation with corresponding lower hippocampal Slc6a4 mRNA in males
			Lower hypothalamic Set7/9, phosphorylated H3S10, and β‐EP, proteins
			Higher hypothalamic G9a, Setdb1, and MeCP2 proteins
			Lower hypothalamic H3K4me3, acetylated H3K9, and POMC mRNA in F1 male and female offspring, and F2 and F3 male offsprings
			Higher hypothalamic H3K9me2, Dnmt1 and POMC promoter methylation in F1 male and female offspring, and F2 and F3 male offsprings
			Hypermethylation of sperm POMC in F1–F3
Chemicals/environmental pollutants	Human/cohort study	BPA exposure in men^284^	Hypomethylation of the sperm LINE‐1 gene
	Human/cohort study	Maternal exposure to organophosphate pesticides and persistent organic pollutants^291^	Decreased adiponectin levels with increasing levels of cord blood *p*,*p*′‐dichlorodiphenyl dichloroethene (DDE) in female offsprings
			Decreased insulin levels with increasing levels of cord blood DDE and polychlorinated biphenyl congener 153 (PCB153) in female offsprings
			Increased insulin levels with increasing levels of maternal urinary Dialkylphosphate (DAP) in female offspring
	Human/cohort study	Association between prenatal prenatal organochlorine compound (OC) concentrations [polychlorinated biphenyls (PCBs), dichlorodiphenyldichloroethylene (DDE), and dichlorodiphenyltrichloroethane (DDT)] and overweight at 6.5 years^279^	Overweight was associated with prenatal PCB and DDE exposure in boys and girls, but with DDT only in boys
	Rat	Maternal dichlorodiphenyltrichloroethane (DDT) exposure during pregnancy^278^	Kidney, prostate and ovary abnormalities in F1 and F3 adults, and mammary tumor development in F1
			Obesity in F3 adults, transmitted via female (egg) and male (sperm) germlines
			Differential methylation changes in multiple sperm DMRs of F3 generation, including those of obesity‐related genes
	Rat	Maternal exposure to Plastics Derived Endocrine Disruptors (BPA, DEHP, and DBP) during pregnancy^280^, ^281^, ^282^, ^283^	Increased kidney and prostate disease
			Hypomethylation in sperm Gck promoter
			Reduced testicular size, and serum and testicular testosterone levels in males
			Increased testicular GnRH mRNA and decreased testicular StAR and P450scc mRNA
			Decreased H3 and H3K14 acetylation in the promoter of StAR
			Increased pubertal abnormalities, testis disease, obesity, and ovarian disease (primary ovarian insufficiency and polycystic ovaries) in F3 generation
			Glucose intolerance and insulin resistance in F2 generation
			Downregulation of Gck mRNA, hypermethylation of the hepatic Gck promoter of F2 liver
			Differential methylation of 197 DMRs in gene promoters in F3 sperm epigenome
	Rat	Maternal vinclozolin exposure during pregnancy^286^	Increased blood lipid, prostate, kidney, immune system and testis abnormalities, and mammary tumor development in F1 to F4 generations (e.g., breast)
	Rat	Maternal Dioxin (TCDD) exposure during pregnancy^287^	Increased prostate disease, ovarian primordial follicle loss and polycystic ovary disease
			Increased kidney disease in males, pubertal abnormalities in females, ovarian primordial follicle loss and polycystic ovary disease in F3 generation
			Differential methylation of 50 DMRs in sperm gene promoters of F3 generation
	Rat	Maternal exposure to permethrin and DEET (pesticide and insect repellent mixture) during pregnancy^288^	Increased pubertal abnormalities, testis disease, and ovarian disease (primordial follicle loss and polycystic ovarian disease) in F3 generation
			Differential methylation of 363 DMRs in sperm gene promoters in F3 generation
	Rat	Maternal exposure to methoxyclhor during pregnancy^290^	Increased kidney disease, ovary disease, and obesity in F1 and the F3 generations
			Differential methylation of genome‐wide DMRs in sperm gene promoters of F3 generation
	Rat	Maternal exposure to jet fuel JP‐8 hydrocarbon during pregnancy^292^	Increased primordial follicle loss and kidney, prostate, pubertal and polycystic ovarian abnormalities
			Increased obesity, primordial follicle loss and polycystic ovarian disease in F3 generations
			Differential methylation of 33 DMRs in sperm gene promoters in F3 generation
	Mice	Maternal exposure to tyibutyltin during pregnancy^289^	Increased white adipose tissue depot weights, adipocyte size, and adipocyte number in F1 to F3 generations
		z	Increased reprogramming of multipotent mesenchymal stem cells toward the adipocyte lineage at the expense of bone in F1 to F3 generations
			Upregulation of hepatic genes involved in lipid storage/transport (Fsp27 and FATP), lipogenesis (PPARγ2, SREBP1c, GyK, and FASN), and lipolysis (PPARα and ACOX) with consequently increased hepatic lipid accumulation F1 to F3 generations
	Zebrafish	Maternal BPA exposure during pregnancy^285^	Decreased reproductive performance in F1–F3 females
			Increased heart failure rates and downregulation of 5 genes (myh6, cmlc2, atp2a2b, sox2, and insrb) genes involved in cardiac development in F1 and F2

^a)^ Effects and/or alterations are those reported in F1 generation due to exposure in F0 in comparison to the control group without the exposure, except otherwise stated. BMI: body mass index; CVD: cardiovascular disease; DMR: differentially methylated region; HDL: high density lipoprotein; LDL: low density lipoprotein; ROS: reactive oxygen species.

**Table 3:** Epigenetic alterations due to methyl donors and other micronutrients in humans and animal experimentation

Nutrient	Specie	Experimental setting [reference]	Physiological changes/epigenetic implicationsa)
Methyl donors including folic acid, methionine, choline, betaine, vitamin B	Human	Cross sectional study of the relationship between maternal and cord blood folate and vitamin B12 levels and methylation of IGF2^103^	Higher methylation in IGF2 P3 in maternal blood than in cord blood, and higher IGF2 P2 methylation in cord blood than in maternal blood. P2 and P3 methylation correlated with serum levels of vitamin B12 in mother's blood, but not in cord blood
			P2 methylation correlated with mother's smoking history and weight gain during pregnancy
	Human	Survey of folic acid intake before and during pregnancy using self‐administered questionnaire, and association with methylation levels of IGF2/H19 DMRs in cord blood^104^	Decreased methylation levels at the H19 DMR with increasing folic acid intake before and during pregnancy
			More pronounced changes in males offsprings
	Human	Associations of maternal vitamin B12, betaine, choline, folate, cadmium, zinc and iron periconceptionally and during the second trimester, and Long Interspersed Nuclear Element‐1 (LINE‐1) methylation levels^113^	Higher cord blood methylation in male than female infants
			Higher maternal cadmium was associated with increased maternal blood first trimester LINE‐1 methylation, and decreased cord blood LINE‐1 methylation
			Increased betaine intake induced lower cord blood LINE‐1 methylation levels
			Increased periconceptional choline decreased cord blood LINE‐1 methylation only in male offsprings
	Human	Associations between periconceptional folic acid use and IGF2 methylation in 17 month old offsprings^98^	Higher maternal S‐adenosylmethionine blood levels, increased IGF2 methylation and lower birth weight with folic acid use
	Mice	Folic acid supplementation (0.4 and 2 mg kg^−1^ diet) before mating and during pregnancy^125^	Hypomethylation of fetal gut Slc394a
	Mice	Maternal choline [high (4.95 g kg^−1^), control (1 g kg^−1^) and low (0 g kg^−1^) of choline in diet) supplementation from day 12 to 17 of pregnancy^105^, ^106^	Decreased proliferation of endothelial cells (EC) and number of blood of blood vessels by 25%–32%, with increased EC differentiation by 25% in the control hippocampus compared with the choline supplemented groups
			Increased expression of angiogenic genes (Vegfc *and* Angpt2) in control fetal hippocampus, with increased ANGPT2 protein
			Hypomethylation of the CpG islands in promoter of Vegfc and Angpt2 in choline deficient group compared with control
			Decreased monomethyl‐lysine 9 of H3 (H3K9me1) in the ventricular and subventricular zones (25%), and dimethyl‐lysine 9 of H3 (H3K9me2) in the pyramidal layer (37%) of hippocampus of choline deficient group
			Reduced expression (80%) of hippocampal G9a histone methyltransferase in choline deficient group
			Hypomethylation of H3 upstream of the RE1 binding site in the calbindin 1 promoter
			Hypermethylation of a CpG site within the calbindin1 promoter. Decreased binding of REST to RE1, which recruits G9a by 45%, with consequent increase in expression of calbindin 1 in choline deficient group
	Mice	Maternal folic acid, vitamin B12, betaine and choline supplementation from day for 8 weeks prior to, and throughout pregnancy and lactation^97^, ^101^, ^102^	Increased DNA methylation at the viable yellow agouti (A(vy)) and Axin (Fu) metastable epialleles
			Dampened transgenerational transmission of obesity
	Rat	Maternal choline [high (4.95 g kg^−1^), control (1 g kg^−1^) and low (0 g kg^−1^) of choline in diet) supplementation from day 12 to 17 of pregnancy^107^, ^108^, ^111^, ^121^, ^122^, ^123^	Improved age‐related memory in choline supplemented group
			Increased S‐Adenosylmethionine levels in fetal liver and brain of choline supplemented group
			Increased IGF2 and IGF2R mRNA and protein and acetylcholine release from the frontal cortex and hippocampus of choline supplemented group
			Increased expression of calcium/calmodulin (CaM)‐dependent protein kinase (CaMK) I in the cortex and transcription factor Zif268/EGR1 in the cortex and hippocampus, and reduced expression of CaMKIIbeta, protein kinase Cbeta2, and GABA(B) receptor 1 isoforms c and d in the hippocampus
			Increased global DNA methylation and overexpression of Dnmt1 mRNA in liver and brain of choline deficient group
			Hypermethylation of Igf2 DMR, increased hepatic Igf2 mRNA levels and hypomethylation of a CpG site within the Dnmt1 locus in fetal liver of the choline deficient group
			Decreased brain and liver Dnmt3a and methyl CpG‐binding domain 2 (Mbd2) protein, and cerebral Dnmt3l in the choline deficient group
			Increased DNA methylation of the G9a and Suv39h1 genes, and with consequent decreases in mRNA and protein expression of G9a and Suv39h1 histone methyltransferases
			Decreased H3K9Me2 and H3K27Me3, and increased H3K4Me2 in choline‐deficient group
			Reduced tumor growth rate in 7,12‐dimethylbenz[alpha]anthracene induced mammary tumors in choline supplemented group
			Increased expression of genes that confer favorable breast cancer outcomes (Klf6, Klf9, Nid2, Ntn4, Per1, and Txnip) and decreased expression of those associated with aggressive disease (Bcar3, Cldn12, Csf1, Jag1, Lgals3, Lypd3, Nme1, Ptges2, Ptgs1, and Smarcb1) in choline supplemented group
			Increased DNA methylation of the tumor suppressor gene, stratifin, with corresponding decrease in expression of its mRNA and protein in mammary tissue of choline supplemented group
	Rat	Prenatal exposure to low (0.59 mmol^−1^ kg d^−1^) or high (3.46 mmol^−1^ kg d^−1^) choline on days 11–18 of pregnancy^109^	Offsprings of choline supplemented group had increased levels of hippocampal BDNF, NGF, and IGF‐1. Choline supplementation also caused less seizure‐induced hippocampal neurodegeneration, dentate cell proliferation, hippocampal GFAP mRNA expression levels, prevented the loss of hippocampal GAD65 protein and mRNA expression, and altered growth factor expression patterns
	Rat	Third trimester ethanol (5.25–6 g kg^−1^ d^−1^) exposure with or without choline supplementation (100–250 mg kg^−1^ d^−1^)^110^, ^112^, ^114^, ^128^	Choline supplementation enhanced offsprings' ethanol induced learning and memory deficits
			Choline attenuated alcohol induced decreases in birth and brain weight, incisor emergence, and behavioral deficits
			Choline supplementation attenuated ethanol‐induced suppression of hypothalamic H3K4me3, Set7/9, acetylated H3K9, phosphorylated H3S10, β‐EP, and POMC mRNA, and increases in H3K9me2, G9a, Setdb1, Dnmt1, MeCP2, POMC gene methylation
	Piglet	Betaine supplementation during pregnancy (3 g kg^−1^ diet) compared with control (no betaine)^115^, ^124^	Betaine supplementation increased serum and hepatic betaine contents, and expression of hepatic methionine metabolic enzymes
			Increased serum concentrations of lactic acid and glucogenic amino acids, including serine, glutamate, methionine and histidine
			Increased hepatic glycogen content, PEPCK1 enzyme activity, protein expression of gluconeogenic enzymes (pyruvate carboxylase, phosphoenolpyruvate carboxykinase 1 and 2, and fructose‐1, 6‐bisphosphatase)
			Reduced hepatic expression of cell cycle regulatory genes, cyclin D2 (CCND2) and presenilin1 (PSEN1)
			Reduced hepatic expression of STAT3, phosphorylation at Tyr705 and Ser727 residues, and STAT3 binding to the CCND2 and PSEN1 promoters
			Reduced STAT3 upstream kinases (phospho‐ERK1/2, phospho‐SRC and phospho‐JAK2)
			STAT3 DNA hypermethylation, and increased H3K27me3, EZH2 and miR‐124a expression, and H3K27me3 on STAT3 promoter
			Increased hippocampal expression of IGF2 and its receptors IGF1R and IGF2R, and the downstream extracellular signal‐regulated kinase 1/2. Hypermethylation of the IGF2 DMRs in the hippocampus of betaine group
			DNA hypermethylation and increased H3K27me3 in the promoter of PEPCK1
			DNA hypomethylation and increased H3K4me3 in promoters of PEPCK2 and FBP1
			Decreased expression of two miRNAs (miRNA 184 and miRNA 196b) targeting pyruvate carboxylase and 6 miRNAs (miRNA‐140‐3p, miRNA‐424‐3p, miRNA‐196b, miRNA‐370, miRNA‐30b‐3p and miRNA‐92b‐5p) targeting PEPCK1 in the liver
	Rat	Dietary protein restriction (90 g kg^−1^ protein) with folic acid supplementation (1 or 3 mg kg^−1^) during pregnancy in rats^93^	Folic acid attenuated low protein‐induced increases in Igf2, H19, Dnmt1 and Dnmt3a, and Mbd2 expression in the liver of male offsprings, and hypermethylation of IGF2/H19 DMRs
	Rat	Maternal high fat diet during pregnancy and lactation with or without methyl donors (15 g Choline Chloride, 15 g Betaine, 15 mg Folic acid, 1.5 mg Vitamin B12, 7.5 g l‐methionine, and 150 mg Zinc)^127^	Methyl donors attenuated high fat diet induced increases in weight gain and fat preference in offsprings
			Attenuated increases in DAT mRNA and MOR mRNA in male and female brain, and global hypomethylation in prefrontal cortex
	Rat	Maternal high fat diet during pregnancy and lactation with or without methyl donors (18 mg folic acid, 1.5 mg vitamin B12, 18 g choline, 7.5 mg l‐methionine, 180 mg zinc, 15 g betaine and 0.3 g genistein)^131^	Methyl donors attenuated high fat diet induced offspring excess weight gain, increased adiposity, insulin resistance, lipid profiles, GLP‐1 and leptin
			Reduced high fat diet induced expression of PPARγ, FAS and adiponectin genes, and enhanced expression of high fat diet induced leptin gene suppression in white adipose
			Increased high fat diet induced hypomethylation of PPARγ, FAS, adiponectin and leptin gene promoter in white adipose
	Rat	Vitamin A (4 IU vitamin A g^−1^ diet) supplementation against diet without vitamin A for 10 weeks prior to, and during pregnancy until gestational day 13^126^	Higher cardiac defects in vitamin A‐deficient group
			Higher methylation of GATA‐4 gene and lower expression of GATA‐4 mRNA in embryos of vitamin A‐deficient group
			Upregulation of DNMT1 and downregulation of DNMT3a and DNMT3b expression
Cadmium/iron	Human	Association between maternal iron, zinc and cadmium and offsprings' birth weight and DNA methylation^129^	Increased maternal blood cadmium levels were associated with lower birth weight, and lower offspring methylation at the MEG3 DMR and PEG3 DMR in males and females respectively
			Lower maternal iron and zinc potentiated the cadmium‐induced hypomethylation of on PEG3 and PLAGL1 DNA
Iron	Rat	iron‐restricted diet (3–10 mg kg^−1^ Fe compared with 225 mg kg^−1^ Fe) given to rats 2 weeks prior to and throughout pregnancy + postnatal high fat diet^99^	15% reduction in birth weight
			Severe anemia at birth
			Higher consumption of high fat diet
			Reduced locomotor activity
			Higher obesity rate
			Increased tendency for salt sensitivity and hypertension
	Rat	Iron restriction in drinking water (3 mg L^−1^ ferrous sulfate compared with 250 mg L^−1^) given to pregnant rats^100^	Higher adipose tissue mass
			Increased serum
			glucose, insulin, triglyceride, leptin, TNFα and IL6 concentrations
			Increased oxidative stress
	Rats	Iron deficient diet (4 mg kg^−1^ Fe) from gestational day 2 through postnatal day 7, and thereafter iron‐sufficient diet (200 mg kg^−1^ Fe), with or without choline supplementation (5 g kg^−1^) from gestational day 11 to 18^116^, ^117^, ^118^, ^119^	Iron deficiency induced memory impairments, and suppression of BDNF‐III and ‐IV mRNAs and BDNF protein, 3‐hydroxy‐3‐methylglutaryl CoA reductase, c‐fos, and early growth response gene 1 and 2
			Iron deficiency suppressed hippocampal expressions hypoxia‐inducible factor 1, dual‐specificity phosphatase 4, IGF 2, and myelin basic protein, and Bndf‐IV P4 methylation, H4 acetylation, K4me3, and binding of RNA polymerase II and USF‐1. It increased HDAC1 binding to Bndf‐IV promoter, and K27me3 and K4me1
			Choline supplementation attenuated iron deficiency induced decreases in hippocampal Bndf protein levels, and binding of USF1 to Bndf‐IV promoter of male offsprings
			Choline reversed iron deficiency induced increase in K27me3 and HDAC1, and decrease in K4me3 in promoter of Bndf‐IV of male offsprings
			Choline supplementation reversed iron deficiency induced alterations in hippocampal expression of multiple genes, including those involved in the molecular networks related to autism and schizophrenia
	Rat	Maternal iron deficiency starting 2 weeks before pregnancy, on day 1, on day 7 or on day 14 of gestation (2–6 mg Fe g^−1^ diet against the normal 1000 mg Fe g^−1^ diet)^130^	Reduced offsprings' body weight, serum iron, hemoglobin and core body, temperature, and delayed auditory brain stem responses
	Rat	Maternal Iron restriction starting 1 week before pregnancy and during gestation (3 mg kg^−1^ against the normal 150 mg kg^−1^)^133^	Decreased hemoglobin, red blood cell count, hematocrit, and mean RBC volume compared with controls
			Lower body weight at birth and at 3 months of age
			Elevated systolic blood pressure at 3 months, but improved glucose tolerance
			Lower fasting serum triglyceride
	Rat	Maternal Iron restriction for 4 weeks prior to and during pregnancy (7.5 mg kg^−1^ against the normal 50 mg kg^−1^)^134^	Gestation day 13 male rat embryo showed significant upregulation of 979 genes and downregulation of 1545 genes involved in processes associated with the initiation of mitosis, BAD‐mediated apoptosis, the assembly of RNA polymerase II preinitiation complexes and WNT signaling
			Upregulation of 7 proteins and downregulation of 10 proteins involved in cell proliferation, protein transport and folding, cytoskeletal remodeling and the proteasome complex
	Mice	Maternal alcohol on gestational day 9 of pregnancy (5.8 g kg^−1^ ethanol) with or without methyl supplementation (15 g Choline, 15 g Betaine, 15 mg Folic Acid, 1.5 mg Vitamin B12, 7.5 g l‐methionine, 150 mg Zinc) prior to, and during pregnancy and lactation^120^	Methyl supplementation attenuated alcohol induced decreases in embryonic IGF2 promoter methylation and IGF 2 expression, and decreased mortality, and digit and vertebral malformations
Zinc	Rat	Maternal zinc restriction 2 weeks before and during pregnancy (10 mg kg^−1^ diet against the normal 35 mg kg^−1^)^132^	Lower offspring weight at birth, weaning and 6 months of age
			Increased body fat, and decreased lean mass, fat‐free mass and fasting plasma insulin levels at 6 months of age
			Lower total cholesterol at 6 months
	Mice	Maternal zinc restriction (5.0 µg Zn g^−1^ against the normal 35 µg Zn g^−1^) from gestation day 8 until delivery, cadmium administration to offsprings at 5 weeks (5 mg kg^−1^)^135^	Increased hepatic MT2 mRNA at 5 weeks
			Altered histone modifications in the MT2 promoter on day 1 and at 5 weeks
			Prolonged MTF1 binding to the MT2 promoter region at 5 weeks
	Mice	Maternal zinc restriction from gestation day 7 to birth^136^	Immunodeficiency in F1–F3 offsprings
	Mice	Maternal zinc restriction (8 ppm against the normal 30 ppm) during pregnancy^137^	Increased systolic blood pressure and decreased glomerular filtration rate associated with a reduction in the number and size of nephrons. Activation of renal apoptosis, reduction in catalase activity, glutathione peroxidase activity, and glutathione levels and increased fibrosis and lipid peroxidation end products
	Mice	Maternal preconception (3–5 d) zinc restriction (<1 mg kg^−1^ against the normal 29 mg kg^−1^)^138^	Decreased histone H3K4 trimethylation and global DNA methylation in zinc deficient oocytes, with 3–20 fold increase in transcript abundance of repetitive elements (Iap, Line1, Sineb1, Sineb2), and a decrease in Gdf9, Zp3 and Figla mRNA
			Only 53% (3 d) and 8% (5 d) of zinc deficient mature eggs reached the 2‐cell stage after IVF
			In vivo fertilized 2‐cell embryos cultured in vitro formed fewer (38%) blastocysts compared to control embryos (74%)
			Decreased Igf2 and H19 mRNA in zinc deficient blastocyst
Magnesium	Rat	Maternal magnesium restriction (0.003% against the normal 0.082% magnesium)^139^	Higher hepatic 11β‐hydroxysteroid dehydrogenase‐2 (Hsd11b2) promoter methylation

^a)^ Effects and/or alterations are those reported in F1 generation due to exposure in F0 in comparison to the control group without the exposure, except otherwise stated. DMR: differentially methylated region; RBC: red blood cells.

In addition to nutritional deficiency during intrauterine life, childhood nutrition has also been linked with adult NCCDs.^58^ Nutritional starvation and specific nutrient deficiency (like iron) during intrauterine life has been associated with an increased tendency for becoming obese especially when exposed to overnutrition during childhood.^78^ Thus, while perinatal undernutrition itself predisposes to obesity later in life,^63^, ^64^ the addition of childhood overnutrition and sudden catch‐up growth further complicates the picture.^59^, ^72^, ^79^ Moreover, intrauterine nutritional starvation has been shown to program offsprings to preferentially consume high fat diets in animals^85^, ^89^, ^94^ and humans,^80^ which may explain the reason for the excessive catch‐up growth in childhood in a nutritionally abundant environment for those exposed prenatally to starvation. Additionally, childhood obesity is associated with increased risks of adult NCCDs and adult‐onset obesity (See Table 2 for details), which itself is a strong risk factor for developing NCCDs.^140^

There is now incontrovertible evidence that prenatal famine had huge implications on health of offsprings of those that experienced these events (Table 2). The Dutch famine of 1944–1945 is perhaps the most extensively studied event linking undernutrition to adult risk of NCCD because of the availability of well‐documented data from the time of the famine, in which case parental starvation around the time of conception and early gestation were reported to significantly affect health outcomes in offsprings' adult life; increased risks of type 2 diabetes, cardiovascular diseases, mental health issues, other NCCDs and even death have been documented.^58^, ^60^, ^62^, ^63^, ^64^, ^65^, ^69^, ^70^, ^71^, ^77^, ^141^ Similarly, in the late 19th and early 20th centuries, famine was a common occurrence in Norbotten, a remote part of Sweden that depended on its agricultural practices to feed its population, when harvest was inadequate.^142^ The children and grandchildren of those to starvation at the time have been shown to have an increased risk of mortality from CVD and type 2 diabetes.^74^, ^76^, ^143^ In addition, sudden changes from nutritional overindulgence to starvation or from starvation to overindulgence can also be responsible for some of the epigenetically determined increases in the risks of offsprings' adult NCCDs.^74^, ^75^ Additionally, studies have shown that epigenetic insults that can influence the risk of offsprings' health do not necessarily have to be experienced around the time of gestation but can set in long before conception.^76^, ^144^, ^145^ More recently, evidence from the Chinese famine of 1959–1961, Biafran famine of 1967–1970, and Ukrainian famine of 1932–1933 have shown increased risks of type 2 diabetes, cardiovascular disease, mental health issues, and other chronic diseases (Table 2).^66^, ^67^, ^68^, ^72^, ^73^, ^84^, ^85^ The Irish, Bangladeshi, Finnish, Soviet Union, and the Gambian famine have also been reported.^69^, ^146^, ^147^ It is thus likely that other cases of famine adversely affected the health of offsprings coming from that exposure but lack of reliable documentary data make it hard to link present day NCCDs to those earlier exposures. Besides, Africa, Asia, and other parts of the world are still having problems of malnutrition^148^ and possibly consequent increases in chronic disease burden.^63^

We know that technological advances started around the time of the industrial revolution, but studies did not document the nutrient needs for pregnant mothers and their growing fetuses until much later. Thus, pregnant mothers did not adhere to any recommendations for supplementation for their health and their babies'. Therefore, it is likely that even outside famine, suboptimal nutrition may have affected the health of individuals exposed to prenatal undernutrition even if the signs of reduced birth weight were absent, which may have been the most obvious sign of undernutrition in the past.^63^, ^64^ Interestingly, even in recent years of advanced healthcare advice, there is evidence that women even in developed countries do not adhere strictly to recommendations around the time of conception that will ensure optimal fetal health and by extension reduce the risk of adult NCCDs later in life (**Table** **4**).^149^, ^150^, ^151^, ^152^, ^153^, ^154^, ^155^, ^156^, ^157^, ^158^, ^159^ The World Health Organization recommends supplementation with several nutrients including folic acid, iron, and others during the time of conception and gestation (**Table** **5**),^160^, ^161^, ^162^, ^163^, ^164^ which many women may fail to take, creating an intrauterine environment that is similar to that of undernutrition in some respects. Moreover, some women may indulge in unhealthy lifestyles including smoking^165^ despite evidence of its adverse effects on fetal health and long term consequences on adult NCCDs. For others, the need to stay slim may be forcing them to unintentionally expose their growing fetuses to the effects of undernutrition, and possibly risk of adult NCCDs.^166^ Sadly, undernutrition is still a prevalent problem worldwide with an estimated one billion suffering from lack of adequate food including pregnant women, through whom growing fetuses are exposed to undernutrition and its attendant risks of diseases later in life.^63^, ^64^ Studies from populations in the Americas (Brazil and Guatemala), South Africa, and Asia (India and the Philippines), where undernutrition was thought to be prevalent in the past indicated strong associations between maternal nutritional status, intrauterine growth restriction, and offsprings' birth weight and BMI at 2 years on one hand with schooling, income, adult BMI, glucose concentrations, and blood pressure. Shorter adult height, less schooling, reduced economic productivity, lower offspring birth weight, high glucose concentrations, high blood pressure, and harmful lipid profiles were more prevalent among those exposed to prenatal malnutrition.^57^ This is a good example of how malnutrition without necessarily widespread famine as that of the Dutch (1944–1945), Chinese (1959–1961), or the Biafran (1967–1970), could influence offsprings' adult risk of NCCDs like type 2 diabetes, cancers, and cardiovascular diseases. Such cases of chronic malnutrition may have been the cases for extended periods of time in many parts of the world, and thus could be influencing the burden of NCCDs to varying degrees. In most of the developing world, many women conceive while they are malnourished. Even if these women end up receiving supplements and nutritional support later in pregnancy, the epigenetic effects of undernutrition can still be traced in their offsprings because early gestation appears to be the most critical time when the effects of undernutrition can induce reprograming events that increase susceptibility to offsprings' adult NCCDs.^64^

**Table 4:** Selected studies highlighting noncompliance to healthy recommendations during pregnancy in different countries

Healthy recommendation [reference]	Observations	Locationa)
Folic acid supplement intake, alcohol consumption, smoking, diet, and physical activity before pregnancy among^149^	Among 238 pregnant women, only 2.9% were taking 400 µg or more of folic acid supplements a day	Southampton, United Kingdom
	Heavy drinking of four or more units of alcohol a week was common	
	74% of those that became pregnant were smokers	
	53% consumed five or more portions of fruit and vegetables a day	
	57% of those who became pregnant had taken any strenuous exercise in the past three months	
Supplementation of 400 µg folic acid daily during the periconceptional period, and exercise recommendation (≥3.5 h a week) by the Danish Health and Medicines Authority^149^, ^150^	Among 22 000 pregnant women, only 22.3% who had planned their pregnancy (13 952) fully complied with the recommendation, while only 13.6% of the overall 22 000 women complied	Copenhagen, Denmark
	Among 7915 pregnant women, only 38% met the recommendation for exercise in early pregnancy	
Dietary iron supplementation during pregnancy^151^	Among 308 pregnant women, 49.7% used iron supplements continuously during the second and third trimesters of pregnancy, while 38.3% reported partial use and 12.0% used no iron supplements	Riyadh, Saudi Arabia
Alcohol intake recommendations during pregnancy^152^, ^153^	Among 837 and 1248 women, 72 and 80% consumed alcohol during pregnancy	Australia
Intermittent presumptive treatment and use of insecticide treated nets during pregnancy^154^	Among 720 pregnant women, 51.6 and 25.9% received the first and second doses of intermittent presumptive treatment, respectively, while 41% slept under insecticide treated nets although only 15.4% used it correctly	Enugu, Nigeria
Dietary behaviors, physical activity, and smoking recommendations^155^	Among 1231 pregnant women in the Latina Gestational Diabetes Mellitus Study, 13% met physical activity guidelines, 19% met fruit/vegetable guidelines, 21% of women smoked, and 1.4% consumed alcohol during pregnancy	Massachusetts, USA
Smoking during pregnancy^156^	The overall prevalence of smoking before pregnancy was 24.7% in 2010, and 12.3% during pregnancy in 11 states of the USA (Alaska, Arkansas, Colorado, Hawaii, Maine, Nebraska, Oklahoma, Utah, Washington, and West Virginia). The prevalence after delivery was 17.2% in 2010	11 states of the USA
Smoking During pregnancy^157^	Among 369 547 pregnant women in Canada, 23% were smokers in 2009–2010, with higher prevalence in the Northern territories (59.3%)	Canada
Perinatal alcohol consumption^158^	Among 1594 pregnant women, 84% had consumed alcohol the year prior to pregnancy (14% considered hazardous consumption) and 6% at least once during pregnancy	Sweden
Perinatal alcohol consumption^159^	Among 1303 pregnant women, two‐thirds and half in the first trimester consumed alcohol over the Department of Health (UK) guidelines of ≤2 units per week before pregnancy	Leeds, UK

^a)^ The observations made from these studies shows that noncompliance to healthy recommendations during pregnancy is not limited to low and middle income countries but that even women in the developed countries mostly do not adhere to those recommendations that will ensure healthy growth of their fetus.

**Table 5:** World Health Organization recommended dietary allowances (RDA) of micronutrients during pregnancy

Nutrient supplementation [reference]	RDA
Folic acid^160^	400 µg of folic acid throughout pregnancy for prevention of neural tube defects, congenital birth defects and possibly risk of adult chronic disease
Iron^160^	30–60 mg of elemental iron throughout pregnancy for prevention of anemia
Vitamin A^161^	Up to 10 000 IU vitamin A (daily dose) OR Up to 25 000 IU vitamin A (weekly dose) for a minimum of 12 weeks during pregnancy until delivery where deficiency is a public health problem for prevention of night blindness
Vitamin D^162^	Insufficient evidence for recommendation
Iodine^163^	250 µg d^−1^ of iodine supplement or 400 mg per year of iodized oil supplement during pregnancy in countries where less than 20% of households have access to iodized salt
Calcium^164^	1.5–2.0 g from 20 weeks of gestation to end of pregnancy in areas where calcium intake is low for prevention of preeclampsia

Additionally, childhood famine has been associated with higher risks of type 2 diabetes, cardiovascular disease, early menopause, breast cancer, and mortality from coronary heart disease.^58^, ^60^, ^70^, ^141^ Conversely, there is increasing evidence of overindulgence in childhood even among those whose parents may have been undernourished due to social mobility and overall demographic changes. These children are at greater risk of adult NCCDs, than those who were exposed to famine alone.^59^ Moreover, those prenatally exposed to the Chinese famine were shown to have higher risks of type 2 diabetes especially in nutritionally rich environments.^72^

### 3.2 Consumption of Western Diets: Effects of Energy Dense Foods

In humans, paternal obesity has been associated with hypomethylation of the MEG3, NDN, SNRPN, and SGCE/PEG10 differentially methylated regions (DMRs) and hypermethylation of the MEG3‐IG and H19 DMRs in the sperm. It has also been associated with hypomethylation of the IGF2, MEST, PEG3, and NNAT DMRs in offsprings' cord blood. Maternal obesity on the other hand has been associated with placental global DNA hypomethylation and hypermethylation of the PLAGL1 DMR and hypomethylation of the MEG3 DMR in cord blood.^165^, ^166^, ^167^, ^168^ Furthermore, in the Dutch hunger cohort, hypomethylation at the IGF2 DMR was reported as one of the underlying factors for the increased risks of several NCCDs among their offsprings,^61^, ^62^ and a similar programing was recently reported for paternal obesity in humans;^169^ paternal obesity was strongly correlated with hypomethylation of the IGF2 DMR and hypermethylation of the H19 DMR, while maternal obesity was correlated with hypermethylation of both IGF2 and H19 DMRs in the cord blood of their offsprings, and thus could partly mediate the multigenerational effects of parental obesity. IGF2/H19 are situated around a cluster of imprinted genes that encode for a fetal and placental growth factor regulating birth weight. The level of methylation of placental IGF2 and H19 DMRs have independently been correlated with circulating third trimester maternal and cord blood IGF2 levels, respectively, and have been shown to modulate fetal growth indices including weight. Additionally, third trimester maternal and cord blood IGF2 levels were also found to be associated with newborn's height. It is thus suggested that IGF2/H19 DMR methylation levels may modulate normal and abnormal fetal and offspring growth and development, with implications for fetal metabolic programing of adult obesity.^171^

Laker et al. demonstrated that maternal high fat diet feeding induced hypermethylation of the muscle peroxisome proliferator‐activated receptor γ coactivator‐1α (Pgc‐1α) in rat, with consequent decreases in the expression of Pgc‐1α and its target genes (Glut4, Cox4, and Cyt c) leading to the development of insulin resistance in offsprings exposed prenatally to high fat diet.^172^ Decreased H3K9Ac and increased H3K9me2 in the white adipose tissue adiponectin promoter, and increased H4K20me in the leptin promoter have also been reported in mice exposed prenatally to high fat diet. These mice preferentially consumed higher calories from 8 weeks onward and had higher body weights by 14 weeks postnatally, high blood pressure, and worsened glucose tolerance by 24 weeks. They also had higher and lower expression of leptin and adiponectin, respectively, in the white adipose tissue, with consequently higher serum triglyceride and leptin levels, and lower adiponectin levels by 12 weeks.^173^ Fullston et al. also showed that the adverse metabolic effects of paternal obesity secondary to high fat diet feeding in mice were transmitted across multiple generations via changes in testes mRNA (414 genes involved in nitric oxide and reactive oxygen species (ROS) pathways, Sertoli cell junction signaling, EIF2 signaling, NF‐*κβ* signaling and inflammatory response, lipid metabolism, and carbohydrate metabolism), sperm and testes microRNA (11 microRNAs mostly involved in metabolic disease, cell death, production of ROS, DNA replication, NF‐κB signaling, p53 signaling, recombination and repair, lipid metabolism, spermatogenesis, and embryonic development) expression, and a 25% reduction in global methylation of germ cell DNA in F0 mice.^174^ Out of the epigenetic alterations observed, those induced by miR‐133b‐3p, miR‐340‐5p, miR‐196a‐5p, and miR‐205‐5p in the testes and sperm of F0 mice exposed to high fat diet were likely the most significant affecting 4 mRNAs (Glt28D2, Hmgb1, Lrrtm3, and Smarce1). In this study, high fat diet feeding increased paternal obesity, which resulted in increased obesity and insulin resistance in both female and male F1 offspring although the risk in F2 was higher in F2 sons whose F1 mothers were exposed prenatally to high fat diet.^174^ The effects of high fat diet feeding may also be mediated via changes in DNA methylation patterns of oocyte metabolism‐related genes;^175^ maternal high fat diet increased DNA methylation of oocyte leptin promoter and decreased that of Ppar‐α promoter, with a corresponding change in methylation levels in the liver (increased leptin and decreased Ppar‐α promoter methylation) and oocyte (increased methylation of Ppar‐α promoter) of F1 female offspring exposed prenatally to high fat diet, and a corresponding change in the hepatic expression of Lep and Ppar‐α. High fat diet feeding and obesity were demonstrated to cause global DNA hypomethylation in the placenta of mice, with additional differential methylation of placental Igf2r regions that serve as binding sites for important transcription factors, including Pax4, Smarca3, Vbp, Pax6, Yy1, Oct1, Nrf2/Arp, Ppar/Rxr, Egr3, Rxr, Mzf1, Sry/Sox9, Gcm1, Stat6, Nudr/Deaf‐1. This consequently modified the expression of genes involved in regulation of placental metabolism including Dio3, Rtl1, Dlk1, Slc22a1, Slc22a2, Slc22a3 especially in the female placenta.^170^ These data and those from other animal studies^127^, ^131^, ^143^, ^176^, ^177^, ^178^, ^179^, ^180^, ^181^, ^182^, ^183^, ^184^, ^185^, ^186^, ^187^, ^188^, ^189^, ^190^, ^191^, ^192^, ^193^, ^194^, ^195^, ^196^, ^197^, ^198^, ^199^, ^200^ mirror those of human trials (see Table 2 for details). However, it is noteworthy that the above studies on obesity were done in humans while the high fat diet feeding studies were done in animals. Thus, the obesity studies have shown very different findings from those of the high fat diet possibly because high fat diet feeding does not only induce obesity but leads to other metabolic abnormalities. Thus, we have reason to believe that these effects are truly different, and studies may have to be conducted simultaneously in future studies to fully unravel the differences and/or similarities in epigenetic outcomes between obesity and high fat diet feeding.

Recommendations focusing on calorie intake often emphasize the need to restrict calories at every stage of development from childhood to adult life. Complying with these recommendations has been shown to favorably reduce the risk of obesity, which is rapidly increasing children and has been blamed on excess calorie intake,^201^, ^202^ although an adverse intrauterine environment may program for childhood obesity and earlier onset of NCCDs^58^, ^59^ by epigenetic programing involving several genes, and may even determine the preference for high energy foods in children thus leading to overnutrition and obesity.^203^, ^204^, ^205^ Increased DNA methylation of the CASP10, CDKN1C, EPHA1, HLADOB3, IRF5, MMP9, MPL, NID1, retinoid X receptor‐α, IGF2/H19 and POMC, and lower methylation of LINE1 in childhood have been shown to impact on gene expression and strongly predict childhood obesity.^206^, ^207^, ^208^, ^209^, ^210^, ^211^ Interestingly, both hypo‐ and hypermethylation of the TACSTD2 gene at birth have been shown to influence expression of the gene and predict childhood obesity (Table 2).^211^ On the other hand, childhood overnutrition increases the risk of developing obesity in childhood and adult life, although the exact epigenetic mechanisms involved are still the subject of debate.^212^

Changes in the gut microbiota induced by western type diets have been shown to alter metabolites' absorption, some of which have direct consequences for fetal programing.^213^ Thus, high fat diets consumed during pregnancy may alter gut microbiota of the pregnant women and affect nutrient availability to the placenta and growing fetus, with resultant implications on epigenetic programing of adult NCCDs later in life. Overall, consumption of western type energy dense foods may be leading to multigenerational programing of obesity.

### 3.3 Consumption of Fruits and Vegetables

Consumption of vegetables, fruits and other nutritious plants, which may have helped in preventing many diseases among the preindustrial humans has fallen drastically in recent periods (Table 1).^28^, ^44^, ^46^, ^48^ The importance of fruits and vegetables is reflected in their inclusion in several recommendations on NCCDs prevention. In fact, their consumption may have epigenetic implications for the transmission of disease risk across multiple generations. Consumption of lower amounts of fruits and vegetables is associated with lower levels of serum folate, minerals, and other vitamins,^214^, ^215^ which are methyl donors that can influence epigenetic programing during intrauterine life. For example, the metabolism of folate, choline, betaine, and other B vitamins generates S‐adenosyl methionine and can ultimately influence DNA and histone methylation (Table 3) by changing the availability of methyl groups to DNA and histone methyltransferases.^96^ Supplementation with methyl donors in the agouti viable yellow (Avy) mice dampened the transgenerational transmission of obesity independent of epigenetic changes at the Avy locus.^97^ IGF DMR hypermethylation may have been involved since prenatal exposure to folic acid, a methyl donor, has been associated with higher levels of maternal S‐adenosylmethionine in the blood, lower birth weight, and higher levels of methylation at the IGF2 DMR in their 17‐month‐old children,^98^ which is associated with a reduced risk for adult NCCDs^61^, ^62^ possibly through regulating DNA and histone methylation levels and intrauterine fetal growth and development for optimal survival, with consequently altered expression of genes that adversely affects postnatal life.^31^, ^171^ Reduced methyl donor availability in pregnant women in the Gambia during the rainy season when harvests were not yet ready was correlated with maternal obesity and was shown to significantly amplify the methylation levels of metastable epialleles (BOLA3, LOC654433, EXD3, ZFYVE28, and ZNF678) in offsprings' lymphocytes and hair follicles.^81^

Prenatal supplementation with folate, a methyl donor, has long been reported to reduce the risk of pregnancy related complications like spontaneous abortions and anemia, and offsprings' congenital diseases like neural tube defect,^172^ and the understanding that it could program the risk of disease in offsprings is an added boost for proponents of such supplementation. Future studies may provide deeper insights into how methyl donor‐altered gene methylation levels transgenerationally influence health outcomes.

### 3.4 Consumption of Highly Processed Low Fiber Grains

Paleolithic diets were predominantly plant based foods that were subjected to very little processing before consumption.^28^, ^44^, ^46^, ^48^ This lack of processing prevented the loss of nutritionally valuable components. Since the advent of food processing techniques, humans have perfected methods of processing their foods in a bid to preserve them for longer duration.^216^ Some of these processing techniques, however, have proven detrimental not only in removing beneficial nutrients but also adding unwanted substances and/or products that may cause harm to consumers (**Table** **6**).^217^, ^218^, ^219^, ^220^ Moreover, diets with high contents of saturated and trans fats, and low fiber content, which often result from food processing, have been linked with development of NCCDs like type 2 diabetes and CVDs.^35^, ^221^ Also, whole grains and other plant foods have high amounts of dietary fiber, which are often removed to enhance palatability and chewiness, thereby leading to lower amounts of dietary fiber in the processed products.^217^

**Table 6:** Some changes introduced by food processing techniques

Beneficial change	Harmful changea)
Increased bioavailability of bioactive compounds due to better release from food matrix, e.g., during cooking	Introduction of trans fatty acids during preparation of fast foods and other commercial junk foods
Improvement of antioxidant properties of naturally occurring compounds, e.g., oxidation of polyphenols during storage increasing their bioactivity	Removal of bioactive‐rich bran layer (including minerals, vitamins and other functional compounds) of grains during polishing
Formation of novel compounds having antioxidant activity (i.e., Maillard reaction products)	Loss of naturally occurring antioxidants, e.g., ascorbic acid during cooking and pasteurization of fruits and vegetables
Increased bioactivity due to synergistic action of compounds from different sources in a single product	Formation of novel compounds having prooxidant activity (i.e., Maillard reaction products)
	Interactions among different compounds mixed in a single product(e.g., lipids and natural antioxidants, lipids and Maillard reaction products)
	Decreased bioactivity due to competitive inhibition of beneficial action when compounds from different sources are mixed in a single product

^a)^ Source: Refs. 217, 218, 219, 220.

Until recent decades, most people consumed unrefined grains like rice, because it was expensive to process the grains to remove the outer layers. Recent advances in processing technologies have however made it easier and more affordable to mill grains. White rice and other polished grains are therefore more accessible, although this has been associated with development of NCCDs like type 2 diabetes and CVD. In particular, white rice is the staple food and main energy source for over half the world's population.^222^ It is likely however that ancestral experiences transmitted epigenetically or energy demands of these people in LMIC is what has determined their taste preferences for energy dense carbohydrate foods like white rice.^89^, ^203^ Sadly, however, white rice has severally been linked to the development of type 2 diabetes, and the risks were particularly more profound among Asians, where white rice consumption is the highest around the world.^223^, ^224^, ^225^ This energy dense grain has high glycemic index and thus leads to higher glycemic and insulin responses following consumption, with increased risks of developing type 2 diabetes and CVDs.^35^, ^222^, ^223^, ^224^, ^225^

The incidence and prevalence of type 2 diabetes is projected to increase mostly in the LMIC by 2040, where coincidentally white rice is the major staple food even among children. In fact, the burden of type 2 diabetes and other NCCDs have already reached alarming proportions in these countries, and although a direct link has not been made, excessive white rice consumption may be a contributing factor toward epigenetic programing of the fetus during intrauterine life.^226^ The epigenetic reprograming that leads to increased risk of type 2 diabetes and possibly other NCCDs in successive generations may not be a direct effect of the white rice per se but a consequence of the metabolic derangements induced by the long term consumption of a high glycemic index diet like white rice; feeding of dams with white rice during the period of gestation and lactation did not produce significant perturbations in metabolic or transcriptomic parameters in their offsprings (unpublished data); however, feeding of white rice for 8 weeks to female rats prior to pregnancy and throughout pregnancy and lactation showed significant differences in metabolic indices related to insulin resistance and insulin signaling genes (hepatic, adipose, and muscle) in comparison to brown rice feeding even when the offsprings were only exposed to normal rodent chow.^226^ Thus, it was hypothesized that consumption of white rice may also be contributing to the growing burden of type 2 diabetes and other metabolic perturbations in the LMICs where white rice is heavily consumed as staple food. In this study, the effects of white rice were only demonstrated in first generation offspring, and future studies will give more insights into how such perturbation affect successive generations. The translational implication of this is that because white rice consumption often starts at very young ages in communities where it is the staple food, children who may have been adversely influenced by their parents' consumption of white rice will start their lives at a disadvantaged point, and their consumption of white rice will further program their bodies to develop type 2 diabetes, and the cycle continues.

The link between whole grain and dietary fiber consumption and health outcomes of offsprings is thought to be mediated via alterations in the gut microbiome. There is growing evidence that the microbes that colonize the gut play significant roles in determining nutrient retrieval from foods through influencing the digestion of consumed foods.^211^ Consumption of higher amounts of dietary fiber has been linked with a distinct gut microbiome often leading to production of higher amounts of butyrate, which is a histone deacetylase inhibitor that could potentially induce epigenetic modifications.^227^, ^228^ There is evidence that short chain fatty acids (SCFA) like butyrate can be absorbed after production in the gut,^229^ thereby potentially reaching the placenta and growing fetus in pregnant women to induce placental histone modifications and possibly other epigenetic effects^230^ and fetal epigenetic reprograming with consequently increased risk of adult NCCDs.^231^ In general, dietary factors can influence the gut microbiota through promoting the preferential growth of some microbiota over others, with resultant production of metabolites such as SCFA and methyl donors that circulate through the blood to reach their target organs and induce DNA methylation changes or histone modifications. The epigenetic programing of specific genes by these metabolites can then influence disease outcomes mediated via abnormal gene regulation.^232^

### 3.5 Technological Advances and Physical Activity Levels

The effects of increasing physical inactivity can be transmitted across several generations.^50^, ^51^, ^233^, ^234^, ^235^ Physical inactivity correlates strongly with both childhood and adult obesity, and obese individuals are more likely to overindulge in western style energy dense foods. Already, we have described how parental obesity programs the offspring to develop NCCDs later in life through epigenetic modulatory changes that affect germ line and placental methylation levels,^167^, ^168^, ^169^, ^175^, ^236^ and gut microbiota.^213^ Increasing physical activity levels have also been shown to induce various epigenetic modifications beneficial to health (Table 2). Moreover, in pregnant rats, exercise was shown to attenuate the high fat diet‐induced hypermethylation of the muscle Pgc‐1α gene and other associated metabolic perturbations in their offsprings.^172^ In humans, six months of exercise induced extensive changes in methylation levels of 7663 genes, including 18 obesity‐related genes.^233^ These and other epigenetic alterations induced by exercise may be the basis for the reduced risk of obesity in children of women who exercise during pregnancy.^51^ Furthermore, exercise is known for reducing weight, and weight loss has important implications on DNA methylation levels,^237^, ^238^, ^239^, ^240^, ^241^ which may be mediated via changes in germ cell methylation levels (Table 2).^234^ Overall, the declining levels of physical activity and increasing sedentary lifestyles are likely contributing factors to the growing burden of global NCCDs across successive generations since evidence exists of the transgenerational effects of obesity and exercise.^50^, ^51^, ^174^, ^242^ However, the understanding of the underlying mechanisms is still limited and future studies are needed to unravel this.

Evolutionary evidence indicates that humans now have higher BMIs than at any point in history, and technological advances that have made humans less active over time have contributed immensely in this regard. Physical activity that was the norm throughout the paleolithic through to the preindustrial periods started to decline with the industrial revolution.^243^ Humans now have machines that have taken over their choirs from households ones to industrial activities involving heavy machinery. Cars and motorcycles have overtaken the use of other more manual methods of transportation. Office jobs that demand siting for long hours have also replaced farming and other physically demanding jobs. Long viewing hours in front of television also make people more sedentary. These phenomena have contributed to the growing burden of obesity, which is a strong determinant for the development of type 2 diabetes, cardiovascular disease and other NCCDs.^244^, ^245^ Similarly, obese individuals can program the health of their offsprings leading to transmission of increased risk of NCCDs through successive generations, while exercise and weight loss can reduce the risks of and/or reverse NCCDs.^23^, ^28^, ^243^, ^246^ The level of physical activity of the hunter gatherer humans was partly the reason why they remained healthy in addition to the healthy dietary choices. In fact, O'keefe et al.^247^ had suggested that adopting a similar level of physical activity to that of the hunter gatherers throughout the entire life of an individual, involving light‐to‐moderate activity targeting an energy expenditure of between 800 and 1200 kcal per day with occasional bursts of high intensity exercise two to three times per week, for at least 20–30 min per session in a natural setting while socializing could dramatically reduce the harmful effects of obesity. This could potentially benefit subsequent generations by programing for a healthier epigenome.

### 3.6 Smoking, Alcohol, Pollutants, and Other Environmental Chemicals

During the preindustrial periods, there were indications that some humans chewed tobacco, but widespread smoking as we know it today may have only started fairly recently. There was an explosion of smoking in the 19th century that quickly spread around the world, which was driven largely by a need for social acceptance.^45^ Increasing awareness, exorbitant taxes and other public health measures have help to reduce smoking rates especially in some developed nations. About 54% of men and 25% of women were smokers in 1955, but by 1995, these rates had dropped to 28% in men and 23% in women.^28^ However, the rates are still high around the world mostly in LMICs, and in some countries smoking remains the most important preventable cause of NCCDs.^248^ The hazards of smoking to the individual's health are now well acknowledged, and in addition second hand smoke is believed to have adverse health consequences on those who do not smoke.^249^ Already, it is well‐documented that smoking during pregnancy directly affects the growing fetus adversely including the risk of low birth weight and increased risk for NCCDs as a result of the toxic effects of the chemicals present in tobacco, but more recent evidence points to contributions of epigenetic alterations (Table 2).^250^ Thus, not only are the constituents of burnt tobacco directly responsible for the adverse effects of smoking, epigenetic alterations induced by the chemicals released has now been shown to underlie some of the smoking‐associated risks of developing NCCDs.^251^ Interestingly, these epigenetic alterations may be heritable, and thus, paternal or maternal preconceptional history of smoking may therefore affect the health of offsprings later in adulthood.

Joehanes et al.^252^ had demonstrated, by analyzing over 16 000 blood samples from smokers, former smokers, and nonsmokers, that tobacco smoke induced long term lymphocyte DNA methylation changes in over 2600 CpG sites annotated to 1405 genes affecting pulmonary function, and risks of cancers, inflammatory diseases, and heart disease, which lasted over 30 years, although quitting was shown to reverse some of these effects within five years. Thus, such long lasting changes may underlie some of the harmful multigenerational effects of smoking. In addition to its long term epigenetic effects, maternal and paternal history have been associated with extensive placental and cord blood DNA methylation changes that are thought to mediate some of the harmful multigenerational effects of smoking (Table 2).^103^, ^253^, ^254^, ^255^, ^256^, ^257^, ^258^, ^259^, ^260^, ^261^ However, the most compelling evidence for the contribution of smoking to epigenetic modulation of disease risk is that from the Avon Longitudinal Study of Parents and Children (ALSPAC) cohort in which fathers who had started smoking around the prepubertal age of 11 had male offsprings with significantly higher BMIs by age 9. The implication is that these obese boys were at increased risk of adult obesity and other obesity‐associated health problems including many NCCDs due to lifestyle choices of their parents. Epigenetic imprinting of genes in the Y chromosome around the time of puberty was thought to be responsible,^142^ and changes in methylation levels of germ line or placenta may propagate the effects across generations.^250^, ^262^ Therefore, widespread smoking and exposure to second hand smoke may not only impact on those directly exposed to it, but also the health of successive generations through epigenetic reprograming that alters the growth and risks of diseases in offsprings. There may even be a multiplier effect since offsprings of smokers are more likely to take up smoking,^263^ because then they will already be at an increased risk of smoking‐related NCCDs due to prenatal exposure and their choice of smoking will greatly compound such risks. Such phenomenon may have contributed to the explosion of chronic diseases around the world from the 19th century onward when widespread use of tobacco began.

Similarly, harmful use of alcohol is believed to be a fairly recent phenomenon, because the excessive intake of harmful levels of alcohol that is characteristic of modern times and associated with NCCDs far exceeds what any preindustrial human could have been able to obtain.^45^ Excessive use of alcohol has been shown to cause adverse health effects, and when consumed during pregnancy it can cause fetal alcohol syndrome ranging from reduced birth weight, to impaired cognitive and neuropsychological functions. Increasing body of evidence indicates that epigenetic alterations may underlie the changes characteristic of fetal alcohol syndrome.^264^ Prenatal exposure to alcohol is demonstrated to induce extensive DNA methylation changes in human^265^, ^266^, ^267^ and rodent offsprings (Table 2).^110^, ^112^, ^114^, ^120^, ^128^, ^268^, ^269^, ^270^, ^271^, ^272^ These effects are possibly transmitted via methylation of the paternal germ line,^273^ although alcohol‐induced placental epigenetic changes suggest the involvement of the female germ line.^264^ The rising harmful use of alcohol,^274^ just as tobacco use,^275^ imposes a huge public health burden, and may have contributed in shaping the history of some NCCDs in recent years. Moreover, chronic alcohol use in males has been shown to increase the chances of alcoholism in male offsprings.^276^ Thus, just like tobacco use, children exposed to alcohol prenatally already have epigenetically predetermined increased risks of some NCCDs, and their subsequent use of alcohol will only worsen the problem.

Pollution is another major public health challenge with huge implications on health of many people especially in the LMICs. In a recent report, the WHO estimated that nine out of ten people globally are affected by poor air quality, which is responsible for the deaths of over six million people annually.^277^ These deaths have been attributed to NCCDs like cardiovascular disease, chronic obstructive pulmonary disease, and lung cancer.^261^ The growth of industrial and other commercial activities have greatly contributed to the problem of pollution since the beginning of the industrial revolution, and diseases associated with pollution have since been on the increase in heavily polluted communities. Additionally, environmental pollutants are commonly found in materials used by humans on a daily basis for agricultural and even domestic purposes. The harm associated with such chemicals has been a concern and some known environmental pollutants like dichlorodiphenyltrichloroethane (DDT) have been banned in some countries like the US for their potential health effects, but they are still being used in other countries for agricultural purposes. Interestingly, recent data indicates that prenatal exposure to some of these pollutants could have huge consequences on transgenerational transmission of the risk of NCCDs (Table 2). For example, prenatal exposure to DDT increases the risk of obesity and other NCCDs via epigenetic alterations in both male and female germ lines.^278^, ^279^ Plastic derived compounds bisphenol‐A (BPA), bis(2‐ethylhexyl)phthalate (DEHP), and dibutyl phthalate (DBP) that are available in most homes have also been linked with increased risk of epigenetic transgenerational inheritance of adult NCCDs, which is mediated via changes in DNA methylation in sperm.^280^, ^281^, ^282^, ^283^, ^284^ Other commonly available environmental and air pollutants including pesticides, agricultural chemicals, and others have also been shown to induce extensive epigenetic changes that are inherited transgenerationally with consequent increases in the risk of adult NCCDs.^285^, ^286^, ^287^, ^288^, ^289^, ^290^, ^291^, ^292^

From the beginning of the industrial revolution, humans have undoubtedly been exposed to a wide range of chemicals and environmental pollutants that could have programed the risk of many diseases in different communities. Moreover, at the beginning of the industrial revolution no legislations were available to control the industrial production of many pollutants or the use of chemicals to manufacture materials for domestic use as we have today. Therefore, such exposures could have compounded the human disease history.

## 4 Nature Versus Nurture: How Did We Get Here?

From the foregoing, it can be surmised that the current global burden of NCCDs may have been influenced by epigenetic factors inherited over time. There have been arguments that the inability to effectively curb the growing burden of chronic diseases reflects the failure of national health systems to properly target intervention strategies against traditional risk factors.^10^ However, it is likely that not only are national health systems inappropriately focusing on strategies that do not work but they may have been ineffective all this while because they are neglecting an important variable (ancestral epigenetic influences). Moreover, despite years of extensive studies and recommendations focusing on lifestyle and dietary factors aimed at curbing the growing trends of NCCDs,^2^, ^3^, ^13^, ^23^, ^24^, ^26^, ^35^, ^36^, ^37^, ^38^, ^41^, ^42^ success has been limited, suggesting a need to refocus efforts on other possible scientific facts contributing to the NCCDs burden for effective control.

Intrauterine epigenetic modifications due to nutritional factors are induced in order to optimize survival of the fetus during intrauterine life, but these changes are retained into adult life and when mixed with certain lifestyle factors amplify the risk of adult NCCDs.^31^ In fact, there are indications that these epigenetic influences may be so potent as to modify the penetrance of a genetic mutation,^197^ which in humans may explain the basis for the recent phenomenon observed with “genetic superheroes”;^293^ adults were found not to exhibit disease phenotypes typical of genetic mutations that are completely penetrant for severe childhood Mendelian disorders like cystic fibrosis. Furthermore, the risk of death from coronary heart disease has long been strongly associated with a family history,^294^ but a convincing genetic link has not been found that can appropriately explain these risks. Other NCCDs like type 2 diabetes and some cancers also have a strong family connection but not genetic risk.^295^ Epigenetic links may be the underlying connection; if ancestors of a certain family undergo lifestyles that increase risk of diseases that can especially be amplified by living certain lifestyles, it will appear as if genetics played a role, when in fact it was an epigenetic trait influencing disease risk in the family.

**Figure 1:**
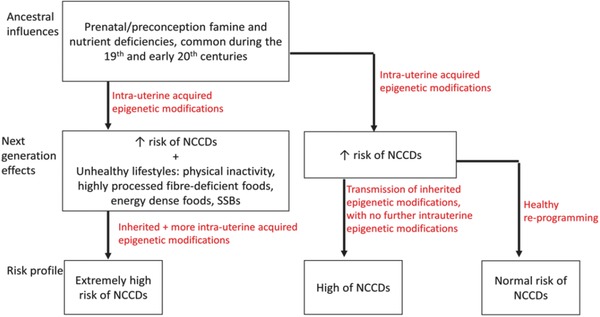
Proposed cumulative effects of epigenetic influences and environmental factors on the chronic disease risk profiles. NCCDs: non‐cmmunicable chronic diseases; SSBs: sugar sweetened beverages.

In the past, it had been suggested that the mismatch between present day diets and ancestral genome may have played a significant role in the growing burden of metabolic diseases.^49^, ^246^ However, epigenetic evidence now suggests that the current burden of NCCDs may have very little to do with such mismatch, but rather the burden of NCCDs as we have them currently may be related to cumulatively inherited epigenetic modifications that have made humans more prone to the NCCDs. We hypothesize, therefore, that ancestral influences including famine and specific nutrient deficiencies, which were common occurrences during the 19th and early 20th centuries may have epigenetically primed our genes, while more recent lifestyle choices including the increased consumption of unhealthy and highly processed energy dense diets, environmental and air pollutants, increasingly sedentary lifestyles, alcohol and tobacco use, and other factors may have further compounded our inherited risks and contributed cumulatively in programing present day humans to have varying risk profiles for NCCDs (**Figure** **1**).

## 5 Future Recommendations

Epigenetic influences on adult risk of chronic diseases may be worse for the future generations if they inherit additional epigenetic alterations due to a combined effect of those from our ancestors and the ones we are currently accumulating through unhealthy lifestyles. If the current trend continues, the future generation may have to pay for the sins of the past and present generations. Thus, there is a need to have deeper understanding on the whole range of epigenetic modifications present and how these influence disease outcome, which is what the Human Epigenome Project (HEP) is aimed at doing;^296^, ^297^ the HEP is a multinational project with a mandate to “identify, catalog, and interpret genome‐wide DNA methylation patterns of all human genes in all major tissues.”^298^ Also, different studies have demonstrated epigenetic effects as a result of specific environmental factors, however, in reality multiple environmental factors are in play at any given point in an individual's life.

Furthermore, future studies must look into the combined epigenetic effects of different environmental factors. Moreover, the effects of different environmental factors may not be additive, but the presence of one environmental factor could modify the effects of another factor. To effectively control the burden of NCCDs, there is a need to target and improve the health of preconception adults (male and female) with a view to achieving a health status that negates adverse intrauterine epigenetics modifications and postnatal health outcomes. Future research into the health of individuals needs to be integrated with not only their lifestyle choices but also those of their ancestors. Thus, the health of individuals must be closely viewed in relation to the health status of their ancestors, while present day humans must take into consideration the health of the future generation in making their lifestyle choices. Also, evidence suggests that epigenetic modifications could be prevented and/or reversed (Tables 2 and 3), indicating that measures can put in place and lifestyles can be modified with a view to improving health outcomes of those at risk of NCCDs due to their previous environmental exposures.

## 6 Conclusions

The world is experiencing an ever increasing burden of NCCDs, which have been traditionally associated with adoption of unhealthy western lifestyle factors including the consumption of energy dense and highly processed foods, physical inactivity, tobacco and alcohol use, exposure to environmental chemicals among others. There is increasing evidence that such environmental factors could induce epigenetic alterations that are transmitted across several generations to program the risk of adult NCCDs in those prenatally exposed. It was argued in this paper that the experiences of our ancestors likely starting around the time of the industrial revolution and those of our immediate parents may have cumulatively programed us to have increased risks of NCCDs, thus the current huge burden and projected increases in the near future. Better understanding of epigenetic influences on chronic disease risk profiles could help in streamlining NCCDs control measures with consequent improved global health. Thus, integrating the health status of individuals with the lifestyle choices of their ancestors will provide deeper insights into individual risks and the overall impact of ancestral influences on the global burden of NCCDs.

## Conflict of Interest

The authors declare no conflict of interest.

## References

[1] J. E. Shaw , R. A. Sicree , P. Z. Zimmet , Diabetes Res. Clin. Pract. 2010, 87, 4. 19896746 10.1016/j.diabres.2009.10.007

[2] World Health Organization , Global action plan for the prevention and control of noncommunicable diseases 2013–2020, http://apps.who.int/iris/bitstream/10665/94384/1/9789241506236_eng.pdf (accessed: April 2017).

[3] World Health Organization , Diet, nutrition, and the prevention of chronic diseases, http://www.who.int/dietphysicalactivity/publications/trs916/en/gsfao_introduction.pdf (accessed: April 2017).

[4] A. Jemal , F. Bray , M. M. Center , J. Ferlay , E. Ward , D. Forman , Ca‐Cancer J. Clin. 2011, 61, 69. 21296855 10.3322/caac.20107

[5] C. J. Murray , A. D. Lopez , N. Engl. J. Med. 2013, 369, 448. 23902484 10.1056/NEJMra1201534

[6] S. Yusuf , S. Reddy , S. Ôunpuu , S. Anand , Circulation 2001, 104, 2746. 11723030 10.1161/hc4601.099487

[7] A. D. Lopez , C. D. Mathers , M. Ezzati , D. T. Jamison , C. J. Murray , Lancet 2006, 367, 1747. 16731270 10.1016/S0140-6736(06)68770-9

[8] A. Boutayeb , S. Boutayeb , Int. J. Equity Health 2005, 4, 2. 15651987 10.1186/1475-9276-4-2 PMC546417

[9] World Health Organization , Cardiovascular diseases fact sheet, http://www.who.int/mediacentre/factsheets/fs317/en (accessed: March 2017).

[10] B. Samb , N. Desai , S. Nishtar , S. Mendis , H. Bekedam , A. Wright , J. Hsu , A. Martiniuk , F. Celletti , K. Patel , F. Adshead , Lancet 2010, 376, 1785. 21074253 10.1016/S0140-6736(10)61353-0

[11] F. B. Hu , Diabetes Care 2011, 34, 1249. 21617109 10.2337/dc11-0442 PMC3114340

[12] Y. Seki , L. Williams , P. M. Vuguin , M. J. Charron , Endocrinolgy 2012, 153, 1031. 10.1210/en.2011-1805 PMC3281534 22253432

[13] A. H. Lichtenstein , L. J. Appel , M. Brands , M. Carnethon , S. Daniels , H. A. Franch , B. Franklin , P. Kris‐Etherton , W. S. Harris , B. Howard , N. Karanja , M. Lefevre , L. Rudel , F. Sacks , L. Van Horn , M. Winston , J. Wylie‐Rosett , N. Karanja , Circulation 2006, 114, 82. 16785338 10.1161/CIRCULATIONAHA.106.176158

[14] G. Danaei , E. L. Ding , D. Mozaffarian , B. Taylor , J. Rehm , C. J. Murray , M. Ezzati , PLoS Med. 2009, 6, e1000058. 19399161 10.1371/journal.pmed.1000058 PMC2667673

[15] K. Charlton , S. Skeaff , Curr. Opin. Clin. Nutr. Metab. Care 2011, 14, 618. 21892078 10.1097/MCO.0b013e32834b2b30

[16] C. Heidemann , M. B. Schulze , O. H. Franco , R. M. van Dam , C. S. Mantzoros , F. B Hu , Circulation 2008, 118, 230. 18574045 10.1161/CIRCULATIONAHA.108.771881 PMC2748772

[17] R. M. van Dam , E. B. Rimm , W. C. Willett , M. J. Stampfer , F. B. Hu , Ann. Intern. Med. 2002, 136, 201. 11827496 10.7326/0003-4819-136-3-200202050-00008

[18] B. J. Gersh , K. Sliwa , B. M. Mayosi , S. Yusuf , Eur. Heart J. 2010, 31, 642. 20176800 10.1093/eurheartj/ehq030

[19] P. A. Heidenreich , J. G. Trogdon , O. A. Khavjou , J. Butler , K. Dracup , M. D. Ezekowitz , E. A. Finkelstein , Y. Hong , S. C. Johnston , A. Khera , D. M. Lloyd‐Jones , Circulation 2011, 123, 933. 21262990 10.1161/CIR.0b013e31820a55f5

[20] F. B. Hu , E. B. Rimm , M. J. Stampfer , A. Ascherio , D. Spiegelman , W. C. Willett , Am. J. Clin. Nutr. 2000, 72, 912. 11010931 10.1093/ajcn/72.4.912

[21] D. Forman , B. E. Bulwer , Curr. Treat. Options Cardiovasc. Med. 2006, 8, 47. 16401383 10.1007/s11936-006-0025-7

[22] K. Teo , S. Lear , S. Islam , P. Mony , M. Dehghan , W. Li , A. Rosengren , P. Lopez‐Jaramillo , R. Diaz , G. Oliveira , M. Miskan , S. Rangarajan , R. Iqbal , R. Ilow , T. Puone , A. Bahonar , S. Gulec , E. A. Darwish , F. Lanas , K. Vijaykumar , O. Rahman , J. Chifamba , Y. Hou , N. Li , S. Yusuf , J. Am. Med. Assoc. 2013, 309, 1613.

[23] N. T. Artinian , G. F. Fletcher , D. Mozaffarian , P. Kris‐Etherton , L. Van Horn , A. H. Lichtenstein , S. Kumanyika , W. E. Kraus , J. L. Fleg , N. S. Redeker , J. C. Meininger , Circulation 2010, 122, 406. 20625115 10.1161/CIR.0b013e3181e8edf1 PMC6893884

[24] D. Kromhout , A. Menotti , H. Kesteloot , S. Sans , Circulation 2002, 105, 893. 11854133 10.1161/hc0702.103728

[25] D. Ornish , L. W. Scherwitz , J. H. Billings , K. L. Gould , T. A. Merritt , S. Sparler , W. T. Armstrong , T. A. Ports , R. L. Kirkeeide , C. Hogeboom , R. J. Brand , J. Am. Med. Assoc. 1998, 280, 2001. 10.1001/jama.280.23.2001 9863851

[26] F. B. Hu , W. C. Willett , J. Am. Med. Assoc. 2002, 288, 2569.

[27] M. J. Stampfer , F. B. Hu , J. E. Manson , E. B. Rimm , W. C. Willett , N. Engl. J. Med. 2000, 343, 16. 10882764 10.1056/NEJM200007063430103

[28] A. R. Walker , B. F. Walker , F. Adam , Nutrition 2003, 19, 169. 10.1016/s0899-9007(02)00948-6 12591555

[29] J. P. Bunker , Int. J. Epidemiol. 2001, 30, 1260. 11821323 10.1093/ije/30.6.1260

[30] J. M. Ordovás , C. E. Smith , Nat. Rev. Cardiol. 2010, 7, 510. 20603647 10.1038/nrcardio.2010.104 PMC3075976

[31] S. H. Zeisel , Am. J. Clin. Nutr. 2009, 89, 1488S. 19261726 10.3945/ajcn.2009.27113B PMC2677001

[32] International Diabetes Federation , Diabetes Atlas, http://www.diabetesatlas.org (accessed: April 2017).

[33] P. Vaughan , L. Gilson , A. Mills , Health Policy Plan. 1989, 4, 97.

[34] D. R. Whiting , L. Guariguata , C. Weil , J. Shaw , Diabetes Res. Clin. Pract. 2011, 94, 311. 22079683 10.1016/j.diabres.2011.10.029

[35] L. A. Bazzano , M. Serdula , S. Liu , J. Am. Coll. Nutr. 2005, 24, 310. 16192254 10.1080/07315724.2005.10719479

[36] J. Lindström , P. Ilanne‐Parikka , M. Peltonen , S. Aunola , J. G. Eriksson , K. Hemiö , H. Hämäläinen , P. Härkönen , S. Keinänen‐Kiukaanniemi , M. Laakso , A. Louheranta , M. Mannelin , M. Paturi , J. Sundvall , T. T. Valle , M. Uusitupa , J. Tuomilehto , Lancet 2006, 368, 1673. 17098085 10.1016/S0140-6736(06)69701-8

[37] G. Li , P. Zhang , J. Wang , E. W. Gregg , W. Yang , Q. Gong , H. Li , H. Li , Y. Jiang , Y. An , Y. Shuai , B. Zhang , J. Zhang , T. J. Thompson , R. B. Gerzoff , G. Roglic , Y. Hu , P. H. Bennett , Lancet 2008, 371, 1783. 18502303 10.1016/S0140-6736(08)60766-7

[38] M. J. Thun , J. O. DeLancey , M. M. Center , A. Jemal , E. M. Ward , Carcinogenesis 2010, 31, 100. 19934210 10.1093/carcin/bgp263 PMC2802672

[39] World Health Organization , Cancer, http://www.who.int/mediacentre/factsheets/fs297/en (accessed: April 2017).

[40] P. Anand , A. B. Kunnumakara , C. Sundaram , K. B. Harikumar , S. T. Tharakan , O. S. Lai , B. Sung , B. B. Aggarwal , Pharm. Res. 2008, 25, 2097. 18626751 10.1007/s11095-008-9661-9 PMC2515569

[41] J. Perk , G. De Backer , H. Gohlke , I. Graham , Ž. Reiner , M. Verschuren , C. Albus , P. Benlian , G. Boysen , R. Cifkova , C. Deaton , Eur. Heart J. 2012, 33, 1635. 22555213 10.1093/eurheartj/ehs092

[42] American Diabetes Association , Diabetes Care 2002, 25, 202.11772917

[43] International Diabetes Federation , Early origins of diabetes, https://www.idf.org/sites/default/files/Policy_Briefing_EarlyOrigins.pdf (accessed: March 2017).

[44] B. Bogin , Growth Horm. IGF Res. 1998, 8, 79. 10990138 10.1016/s1096-6374(98)80027-0

[45] S. B. Eaton , M. Konner , M. Shostak , Am. J. Med. 1988, 84, 739. 3135745 10.1016/0002-9343(88)90113-1

[46] K. O'Dea , Current Problems in Nutrition Pharmacology and Toxicology, John Libbey, London, UK 1988, pp. 26–35.

[47] S. B. Eaton , M. Konner , N. Engl. J. Med. 1985, 312, 283. 2981409 10.1056/NEJM198501313120505

[48] A. G. Henry , A. S. Brooks , D. R. Piperno , J. Human Evol. 2014, 69, 44. 24612646 10.1016/j.jhevol.2013.12.014

[49] J. H. O'Keefe , L. Cordain , Mayo Clin. Proc. 2004, 79, 101. 14708953 10.4065/79.1.101

[50] R. Barrès , J. R. Zierath , Nat. Rev. Endocrinol. 2016, 12, 441. 27312865 10.1038/nrendo.2016.87

[51] T. E. Chalk , W. M. Brown , Epigenomics 2014, 6, 469. 25431939 10.2217/epi.14.38

[52] K. Hill , A. M. Hurtado , R. S. Walker , J. Hum. Evol. 2007 52, 443. 17289113 10.1016/j.jhevol.2006.11.003

[53] E. Jutikkala , M. Kauppinen , Pop. Stud. 1971, 25, 283. 10.1080/00324728.1971.10405803 22070112

[54] NCD Risk Factor Collaboration , Lancet 2016, 387, 1377.27115820

[55] L.P. Palaniappan , M. R. G. Araneta , T. L. Assimes , E. L. Barrett‐Connor , M. R. Carnethon , M. H. Criqui , G. L. Fung , K. V. Narayan , H. Patel , R. E. Taylor‐Piliae , P. W. Wilson , Circulation 2010, 122, 1242. 20733105 10.1161/CIR.0b013e3181f22af4 PMC4725601

[56] J. F. Kurtzke , G. W. Beebe , J. E. Norman , Neurology 1985, 35, 672. 3873023 10.1212/wnl.35.5.672

[57] M. L. V. Pimentel , Arq. Neuropsiquiatr. 2013, 71, 569. 24141432 10.1590/0004-282X20130114

[58] A. C. J. Ravelli , J. H. P. Van der Meulen , C. Osmond , D. J. P. Barker , O. P. Bleker , Arch. Dis. Child. 2000, 82, 248. 10685933 10.1136/adc.82.3.248 PMC1718232

[59] C. G. Victora , L. Adair , C. Fall , P. C. Hallal , R. Martorell , L. Richter , H.S. Sachdev , Maternal and Child Undernutrition Study Group , Lancet 2008, 371, 340. 18206223 10.1016/S0140-6736(07)61692-4 PMC2258311

[60] A. F. van Abeelen , S. G. Elias , P. M. Bossuyt , D. E. Grobbee , Y. T. van der Schouw , T. J. Roseboom , C. S. Uiterwaal , Diabetes 2012, 61, 2255. 22648386 10.2337/db11-1559 PMC3425424

[61] E. W. Tobi , L. H. Lumey , R. P. Talens , D. Kremer , H. Putter , A. D. Stein , P. E. Slagboom , B. T. Heijmans , Human Mol. Gen. 2009, 18, 4046. 10.1093/hmg/ddp353 PMC2758137 19656776

[62] B. T. Heijmans , E. W. Tobi , A. D. Stein , H. Putter , G. J. Blauw , E. S. Susser , P. E. Slagboom , L. H. Lumey , Proc. Nat. Acad. Sci. USA 2008, 105, 17046. 18955703 10.1073/pnas.0806560105 PMC2579375

[63] T. J. Roseboom , R. C. Painter , A. F. van Abeelen , M. V. Veenendaal , S. R. de Rooij , Maturitas 2011, 70, 141. 21802226 10.1016/j.maturitas.2011.06.017

[64] T. Roseboom , S. de Rooij , R. Painter , Early Hum. Dev. 2006, 82, 485. 16876341 10.1016/j.earlhumdev.2006.07.001

[65] R. C. Painter , T. J. Roseboom , P. M. M. Bossuyt , C. Osmond , D. J. P. Barker , O. P. Bleker , Eur. J. Epidemiol. 2005, 20, 673. 16151880 10.1007/s10654-005-7921-0

[66] M. Hult , P. Tornhammar , P. Ueda , C. Chima , A. K. E. Bonamy , B. Ozumba , M. Norman , PloS One 2010, 5, e13582. 21042579 10.1371/journal.pone.0013582 PMC2962634

[67] Y. Y. Chen , L. A. Zhou , J. Health Econ. 2007, 26, 659e681. 17289187 10.1016/j.jhealeco.2006.12.006

[68] C. Huang , M. R. Phillips , Y. Zhang , J. Zhang , Q. Shi , Z. Song , Z. Ding , S. Pang , R. Martorell , Social Sci. Med. 2013, 97, 259. 10.1016/j.socscimed.2012.09.051 PMC3726543 23313495

[69] L. H. Lumey , A. D. Stein , E. Susser , Annu. Rev. Public Health 2011, 32, 237. 21219171 10.1146/annurev-publhealth-031210-101230 PMC3857581

[70] I. Koupil , D. B. Shestov , P. Sparén , S. Plavinskaja , N. Parfenova , D. Vågerö , Eur. J. Epidemiol. 2007, 22, 223. 17436055 10.1007/s10654-007-9113-6

[71] P. Ekamper , F. van Poppel , A. D. Stein , G. E. Bijwaard , L. H. Lumey , Am. J. Epidemiol. 2015, 181, 271. 25632050 10.1093/aje/kwu288 PMC4325678

[72] Y. Li , Y. He , L. Qi , V. W. Jaddoe , E. J. Feskens , X. Yang , G. Ma , F. B. Hu , Diabetes 2010, 59, 2400. 20622161 10.2337/db10-0385 PMC3279550

[73] L. H. Lumey , M. D. Khalangot , A. M. Vaiserman , Lancet Diabetes Endocrinol. 2015, 3, 787. 26342852 10.1016/S2213-8587(15)00279-X

[74] L. O. Bygren , P. Tinghög , J. Carstensen , S. Edvinsson , G. Kaati , M. E. Pembrey , M. Sjöström , BMC Genet. 2014, 15, 12. 24552514 10.1186/1471-2156-15-12 PMC3929550

[75] L. O. Bygren , S. Edvinsson , G. Broström , Am. J. Hum. Biol. 2000, 12, 447. 11534035 10.1002/1520-6300(200007/08)12:4<447::AID-AJHB3>3.0.CO;2-M

[76] L. O. Bygren , G. Kaati , S. Edvinsson , Acta Biotheor. 2001, 49, 53. 11368478 10.1023/a:1010241825519

[77] E. W. Tobi , J. J. Goeman , R. Monajemi , H. Gu , H. Putter , Y. Zhang , R. C. Slieker , A. P. Stok , P. E. Thijssen , F. Müller , E. W. van Zwet , Nat. Commun. 2014, 5, 5592. 25424739 10.1038/ncomms6592 PMC4246417

[78] C. Yajnik , Proc. Nutr. Soc. 2000, 59, 257. 10946794 10.1017/s0029665100000288

[79] B. Fagerberg , L. Bondjers , P. Nilsson , J. Intern. Med. 2004, 256, 254. 15324369 10.1111/j.1365-2796.2004.01361.x

[80] M. M. Perälä , S. Männistö , N. E. Kaartinen , E. Kajantie , C. Osmond , D. J. Barker , L. M. Valsta , J. G. Eriksson , PLoS One 2012, 7, e46139. 23049962 10.1371/journal.pone.0046139 PMC3458835

[81] P. Dominguez‐Salas , S. E. Moore , M. S. Baker , A. W. Bergen , S. E. Cox , R. A. Dyer , A. J. Fulford , Y. Guan , E. Laritsky , M. J. Silver , G. E. Swan , Nat. Commun. 2014, 5, 3746. 24781383 10.1038/ncomms4746 PMC4015319

[82] G. Kaati , L. O. Bygren , S. Edvinsson , Eur. J. Hum. Genet. 2002, 10, 682. 12404098 10.1038/sj.ejhg.5200859

[83] S. E. Moore , T. J. Cole , E. M. Poskitt , B. J. Sonko , R. G. Whitehead , A. M. Prentice , Nature 1997, 388, 434. 9242401 10.1038/41245

[84] M. Q. Xu , W. S. Sun , B. X. Liu , G. Y. Feng , L. Yu , L. Yang , G. He , P. Sham , E. Susser , D. S. Clair , L. He , Schizophr. Bull. 2009, 35, 568. 19155344 10.1093/schbul/sbn168 PMC2669578

[85] D. St Clair , M. Xu , P. Wang , Y. Yu , Y. Fang , F. Zhang , X. Zheng , N. Gu , G. Feng , P. Sham , L. He , J. Am. Med. Assoc. 2005, 294, 557. 10.1001/jama.294.5.557 16077049

[86] A. Erhuma , L. Bellinger , S. C. Langley‐Evans , A. J. Bennett , Br. J. Nutr. 2007, 98, 517. 17442129 10.1017/S0007114507721505 PMC3861785

[87] K. A. Lillycrop , E. S. Phillips , A. A. Jackson , M. A. Hanson , G. C. Burdge , J. Nutr. 2005, 135, 1382. 15930441 10.1093/jn/135.6.1382

[88] G. C. Burdge , J. Slater‐Jefferies , C. Torrens , E. S. Phillips , M. A. Hanson , K. A. Lillycrop , Br. J. Nutr. 2007, 97, 435. 17313703 10.1017/S0007114507352392 PMC2211514

[89] L. Bellinger , C. Lilley , S. C. Langley‐Evans , Br. J. Nutr. 2004, 92, 513. 15469656 10.1079/bjn20041224

[90] N. Peixoto‐Silva , E. D. C. Frantz , C. A. Mandarim‐de‐Lacerda , A. Pinheiro‐Mulder , Br. J. Nutr. 2011, 106, 1364. 21736811 10.1017/S0007114511001735

[91] E. J. Radford , M. Ito , H. Shi , J. A. Corish , K. Yamazawa , E. Isganaitis , S. Seisenberger , T. A. Hore , W. Reik , S. Erkek , A. H. Peters , Science 2014, 345, 1255903. 25011554 10.1126/science.1255903 PMC4404520

[92] P. Y. Chen , A. Ganguly , L. Rubbi , L. D. Orozco , M. Morselli , D. Ashraf , A. Jaroszewicz , S. Feng , S. E. Jacobsen , A. Nakano , S. U. Devaskar , Physiol. Genomics 2013, 45, 565. 23695884 10.1152/physiolgenomics.00034.2013 PMC3727019

[93] L. Gong , Y. X. Pan , H. Chen , Epigenetics 2010, 5, 619. 20671425 10.4161/epi.5.7.12882

[94] C. Jousse , L. Parry , S. Lambert‐Langlais , A. C. Maurin , J. Averous , A. Bruhat , V. Carraro , J. Tost , P. Letteron , P. Chen , R. Jockers , FASEB J. 2011, 25, 3271. 21670064 10.1096/fj.11-181792

[95] E. M. van Straten , V. W. Bloks , N. C. Huijkman , J. F. Baller , H. van Meer , D. Lütjohann , F. Kuipers , T. Plösch , Am. J. Physiol. Regul. Integr. Comp. Physiol. 2010, 298, R275. 19889862 10.1152/ajpregu.00413.2009

[96] O. S. Anderson , K. E. Sant , D. C. Dolinoy , J. Nutr. Biochem. 2012, 23, 853. 22749138 10.1016/j.jnutbio.2012.03.003 PMC3405985

[97] R. A. Waterland , M. Travisano , K. G. Tahiliani , M. T. Rached , S. Mirza , Int. J. Obes. 2008, 32, 1373. 10.1038/ijo.2008.100 PMC2574783 18626486

[98] R. P. Steegers‐Theunissen , S. A. Obermann‐Borst , D. Kremer , J. Lindemans , C. Siebel , E. A. Steegers , P. E. Slagboom , B. T. Heijmans , PloS One 2009, 4, e7845. 19924280 10.1371/journal.pone.0007845 PMC2773848

[99] S. L. Bourque , M. Komolova , K. McCabe , M. A. Adams , K. Nakatsu , Endocrinology 2012, 153, 1174. 22210741 10.1210/en.2011-1700

[100] A. A. Tinkov , E. V. Popova , V. S. Polyakova , A. A. Nikonorov , BioMetals 2014, 27, 293. 24519243 10.1007/s10534-014-9712-0

[101] D. C. Dolinoy , D. Huang , R. L. Jirtle , Proc. Nat. Acad. Sci. USA 2007, 104, 13056. 17670942 10.1073/pnas.0703739104 PMC1941790

[102] R. A. Waterland , D. C. Dolinoy , J. R. Lin , C. A. Smith , X. Shi , K. G. Tahiliani , Genesis 2006, 44, 401. 16868943 10.1002/dvg.20230

[103] Y. Ba , H. Yu , F. Liu , X. Geng , C. Zhu , Q. Zhu , T. Zheng , S. Ma , G. Wang , Z. Li , Y. Zhang , Eur. J. Clin. Nutr. 2011, 65, 480. 21245875 10.1038/ejcn.2010.294 PMC3071883

[104] C. Hoyo , A. P. Murtha , J. M. Schildkraut , R. L. Jirtle , W. Demark‐Wahnefried , M. R. Forman , E. S. Iversen , J. Kurtzberg , F. Overcash , Z. Huang , S. K. Murphy , Epigenetics 2011, 6, 928. 21636975 10.4161/epi.6.7.16263 PMC3154433

[105] M. G. Mehedint , C. N. Craciunescu , S. H. Zeisel , Proc. Nat. Acad. Sci. USA 2010, 107, 12834. 20624989 10.1073/pnas.0914328107 PMC2919920

[106] M. G. Mehedint , M. D. Niculescu , C. N. Craciunescu , S. H. Zeisel , FASEB J. 2010, 24, 184. 19752176 10.1096/fj.09-140145 PMC2797040

[107] V. P. Kovacheva , T. J. Mellott , J. M. Davison , N. Wagner , I. Lopez‐Coviella , A. C. Schnitzler , J. K. Blusztajn , J. Biol. Chem. 2007, 282, 31777. 17724018 10.1074/jbc.M705539200

[108] J. M. Davison , T. J. Mellott , V. P. Kovacheva , J. K. Blusztajn , J. Biol. Chem. 2009, 284, 1982. 19001366 10.1074/jbc.M807651200 PMC2629111

[109] S. J. Wong‐Goodrich , T. J. Mellott , M. J. Glenn , J. K. Blusztajn , C. L. Williams , Neurobiol. Dis. 2008, 30, 255. 18353663 10.1016/j.nbd.2008.01.008 PMC2413180

[110] S. H. Ryan , J. K. Williams , J. D. Thomas , Brain Res. 2008, 1237, 91. 18786517 10.1016/j.brainres.2008.08.048 PMC2646103

[111] V. P. Kovacheva , J. M. Davison , T. J. Mellott , A. E. Rogers , S. Yang , M. J. O'Brien , J. K. Blusztajn , FASEB J. 2009, 23, 1054. 19047067 10.1096/fj.08-122168 PMC2660648

[112] J. D. Thomas , E. J. Abou , H. D. Dominguez , Neurotoxicol. Teratol. 2009, 31, 303. 19616089 10.1016/j.ntt.2009.07.002 PMC2952280

[113] C. E. Boeke , A. Baccarelli , K. P. Kleinman , H. H. Burris , A. A. Litonjua , S. L. Rifas‐Shiman , L. Tarantini , M. Gillman , Epigenetics 2012, 7, 253. 22430801 10.4161/epi.7.3.19082 PMC3335948

[114] R. A. Bekdash , C. Zhang , D. K. Sarkar , Alcohol. Clin. Exp. Res. 2013, 37, 1133. 23413810 10.1111/acer.12082 PMC3659188

[115] D. Cai , Y. Jia , H. Song , S. Sui , J. Lu , Z. Jiang , R. Zhao , PloS One 2014, 9, e105504. 25153319 10.1371/journal.pone.0105504 PMC4143294

[116] P. V. Tran , B. C. Kennedy , Y. C. Lien , R. A. Simmons , M. K. Georgieff , Am. J. Physiol. Regul. Integr. Comp. Physiol. 2015, 308, R276. 25519736 10.1152/ajpregu.00429.2014 PMC4329464

[117] P. V. Tran , B. C. Kennedy , M. T. Pisansky , K. J. Won , J. C. Gewirtz , R. A. Simmons , M. K. Georgieff , J. Nutr. 2016, 146, 484. 26865644 10.3945/jn.115.227561 PMC4763487

[118] P. V. Tran , S. J. Fretham , E. S. Carlson , M. K. Georgieff , Pediatr. Res. 2009, 65, 493. 19190544 10.1203/PDR.0b013e31819d90a1 PMC2715440

[119] B. C. Kennedy , J. G. Dimova , A. J. Siddappa , P. V. Tran , J. C. Gewirtz , M. K. Georgieff , J. Nutr. 2014, 144, 1858. 25332485 10.3945/jn.114.198739 PMC4195423

[120] C. Downing , T. E. Johnson , C. Larson , T. I. Leakey , R. N. Siegfried , T. M. Rafferty , C. A. Cooney , Alcohol 2011, 45, 65. 20705422 10.1016/j.alcohol.2010.07.006 PMC2997169

[121] T. J. Mellott , M. T. Follettie , V. Diesl , A. A. Hill , I. Lopez‐Coviella , J. K. Blusztajn , FASEB J. 2007, 21, 1311. 17264169 10.1096/fj.06-6597com

[122] T. J. Mellott , N. W. Kowall , I. Lopez‐Coviella , J. K. Blusztajn , Brain Res. 2007, 1151, 1. 17399691 10.1016/j.brainres.2007.03.004 PMC1952662

[123] I. Napoli , J. K. Blusztajn , T. J. Mellott , Brain Res. 2008, 1237, 124. 18786520 10.1016/j.brainres.2008.08.046

[124] X. Li , Q. Sun , X. Li , D. Cai , S. Sui , Y. Jia , H. Song , R. Zhao . Eur. J. Nutr. 2015, 54, 1201. 25410747 10.1007/s00394-014-0799-4

[125] J. A. McKay , Y. K. Wong , C. L. Relton , D. Ford , J. C. Mathers , Mol. Nutr. Food Res. 2011, 55, 1717. 21770049 10.1002/mnfr.201100150

[126] Y. Feng , L. Z. Zhao , L. Hong , C. Shan , W. Shi , W. Cai , J. Nutr. Biochem. 2013, 24, 1373. 23333085 10.1016/j.jnutbio.2012.11.005

[127] J. Carlin , R. George , T. M. Reyes , PLoS One 2013, 8, e63549. 23658839 10.1371/journal.pone.0063549 PMC3642194

[128] J. D. Thomas , N. M. Idrus , B. R. Monk , H. D. Dominguez , Birth Defects Res. A. Clin. Mol. Teratol. 2010, 88, 827. 20706995 10.1002/bdra.20713 PMC3677823

[129] A. C. Vidal , V. Semenova , T. Darrah , A. Vengosh , Z. Huang , K. King , M. D. Nye , R. Fry , D. Skaar , R. Maguire , A. Murtha , J. Schildkraut , S. Murphy , C. Hoyo , BMC Pharmacol. Toxicol. 2015, 16, 20. 26173596 10.1186/s40360-015-0020-2 PMC4502530

[130] C. Mihaila , J. Schramm , F. G. Strathmann , D. L. Lee , R. M. Gelein , A. E. Luebke , M. Mayer‐Pröschel , PLoS One 2011, 6, e17483. 21423661 10.1371/journal.pone.0017483 PMC3057971

[131] F. Jiao , X. Yan , Y. Yu , X. Zhu , Y. Ma , Z. Yue , H. Ou , Z. Yan , J. Nutr. Biochem. 2016, 34, 42. 27183114 10.1016/j.jnutbio.2016.04.005

[132] I. J. Padmavathi , Y. D. Kishore , L. Venu , M. Ganeshan , N. Harishankar , N. V. Giridharan , M. Raghunath , Exp. Physiol. 2009, 94, 761. 19251982 10.1113/expphysiol.2008.045856

[133] R. M. Lewis , C. J. Petry , S. E. Ozanne , C. N. Hales , Metabolism 2001, 50, 562. 11319718 10.1053/meta.2001.22516

[134] A. Swali , S. McMullen , H. Hayes , L. Gambling , H. J. McArdle , S. C. Langley‐Evans , PloS One 2012, 7, e48133. 23110188 10.1371/journal.pone.0048133 PMC3482177

[135] H. Kurita , S. Ohsako , S. I. Hashimoto , J. Yoshinaga , C. Tohyama , J. Nutr. Biochem. 2013, 24, 256. 22917840 10.1016/j.jnutbio.2012.05.013

[136] R. S. Beach , M. E. Gershwin , L. S. Hurley , Science 1982, 218, 469. 7123244 10.1126/science.7123244

[137] A. L. Tomat , F. Inserra , L. Veiras , M. C. Vallone , A. M. Balaszczuk , M. A. Costa , C. Arranz , Am. J. Physiol. Regul. Integr. Comp. Physiol. 2008, 295, R543. 18525016 10.1152/ajpregu.00050.2008

[138] X. Tian , F. J. Diaz , Dev. Biol. 2013, 376, 51. 23348678 10.1016/j.ydbio.2013.01.015 PMC3601821

[139] J. Takaya , A. Iharada , H. Okihana , K. Kaneko , Epigenetics 2011, 6, 573. 21406963 10.4161/epi.6.5.15220

[140] M. M. Kelsey , A. Zaepfel , P. Bjornstad , K. J. Nadeau , Gerontology 2014, 60, 222. 24434909 10.1159/000356023

[141] U. G. Kyle , C. Pichard , Curr. Opin. Clin. Nutr. Metab. Care 2006, 9, 388. 16778567 10.1097/01.mco.0000232898.74415.42

[142] J. Cloud , Time Mag. 2010, 18, 49. 20722335

[143] Z. Vucetic , J. Kimmel , K. Totoki , E. Hollenbeck , T. M. Reyes , Endocrinology 2010, 151, 4756. 20685869 10.1210/en.2010-0505 PMC2946145

[144] M. E. Pembrey , L. O. Bygren , G. Kaati , S. Edvinsson , K. Northstone , M. Sjöström , J. Golding , Eur. J. Hum. Genet. 2006, 14, 159. 16391557 10.1038/sj.ejhg.5201538

[145] M. Pembrey , R. Saffery , L. O. Bygren , J. Carstensen , S. Edvinsson , T. Faresjö , P. Franks , J. Å. Gustafsson , G. Kaati , L. H. Lumey , B. Modin , J. Med. Genet. 2014, 51, 563. 25062846 10.1136/jmedgenet-2014-102577 PMC4157403

[146] R. Hernández‐Julián , H. Mansour , C. Peters , Demography 2014, 51, 1775. 25189157 10.1007/s13524-014-0326-5

[147] C. Ó. Gráda , K. H. O'Rourke , Eur. Rev. Econ. Hist. 1997, 1, 3.

[148] Food and Agriculture Organization , The State of Food Insecurity in the World 2015: Strengthening the enabling environment for food security and nutrition, http://www.fao.org/3/a4ef2d16‐70a7‐460a‐a9ac‐2a65a533269a/i4646e.pdf (accessed: March 2017).

[149] V. K. Knudsen , I. Orozova‐Bekkevold , L. B. Rasmussen , T. B. Mikkelsen , K. F. Michaelsen , S. F. Olsen , Public Health Nutr. 2004, 7, 843. 15482608 10.1079/phn2004630

[150] L. Broberg , A. S. Ersbøll , M. G. Backhausen , P. Damm , A. Tabor , H. K. Hegaard , BMC Pregnancy Childbirth 2015, 15, 317. 26614105 10.1186/s12884-015-0756-0 PMC4661949

[151] F. Habib , E. Habib Zein Alabdin , M. Alenazy , R. Nooh , J. Obstet. Gynaecol. 2009, 29, 487. 19697194 10.1080/01443610902984961

[152] J. R. Powers , D. J. Loxton , L. A. Burns , A. Shakeshaft , E. J. Elliott , A. J. Dunlop , Med. J. Aust. 2010, 192, 690. 20565346 10.5694/j.1326-5377.2010.tb03703.x

[153] A. E. Anderson , A. J. Hure , J. R. Powers , F. J. Kay‐Lambkin , D. J. Loxton , BMC Public Health 2012, 12, 777. 22971176 10.1186/1471-2458-12-777 PMC3511880

[154] N. G. Onyeneho , N. Idemili‐Aronu , I. Okoye , C. Ugwu , F. U. Iremeka , Maternal Child Health J. 2014, 18, 1169. 10.1007/s10995-013-1347-1 24043556

[155] A. Gollenberg , P. Pekow , G. Markenson , K. L. Tucker , L. Chasan‐Taber , Am. J. Clin. Nutr. 2008, 87, 1844. 18541576 10.1093/ajcn/87.6.1844

[156] V. T. Tong , P. M. Dietz , B. Morrow , D. V. D'Angelo , S. L. Farr , K. M. Rockhill , L. J. England , MMWR Surveill. Summ. 2013, 62, 1. 24196750

[157] Y. Cui , S. Shooshtari , E. L. Forget , I. Clara , K. F. Cheung , PloS One 2014, 9, e84640. 24416257 10.1371/journal.pone.0084640 PMC3885577

[158] J. Skagerström , S. Alehagen , E. Häggström‐Nordin , K. Årestedt , P. Nilsen , BMC Public Health 2013, 13, 780. 23981786 10.1186/1471-2458-13-780 PMC3765772

[159] C. Nykjaer , N. A. Alwan , D. C. Greenwood , N. A. Simpson , A. W. Hay , K. L. White , J. E. Cade , J. Epidemiol. Commun. Health 2014, 68, 542. 10.1136/jech-2013-202934 PMC4033207 24616351

[160] World Health Organization , Guideline: Daily iron and folic acid supplementation in pregnant women, http://www.who.int/nutrition/publications/micronutrients/guidelines/daily_ifa_supp_pregnant_women/en/ (accessed: April 2017). 23586119

[161] World Health Organization , Vitamin A supplementation in pregnant women, http://apps.who.int/iris/bitstream/10665/44625/1/9789241501781_eng.pdf?ua=1&ua=1 (accessed: April 2017).

[162] World Health Organization , Vitamin D supplementation in pregnant women, http://apps.who.int/iris/bitstream/10665/85313/1/9789241504935_eng.pdf?ua=1 (accessed: April 2017).

[163] World Health Organization , Iodine supplementation in pregnant and lactating women, http://www.who.int/elena/titles/guidance_summaries/iodine_pregnancy/en/ (accessed: April 2017).

[164] World Health Organization , Guideline: Calcium supplementation in pregnant women, http://apps.who.int/iris/bitstream/10665/85120/1/9789241505376_eng.pdf (accessed: April 2017). 24006556

[165] H. M. Inskip , S. R. Crozier , K. M. Godfrey , S. E. Borland , C. Cooper , S. M. Robinson , Southampton Women's Survey Study Group , Br. Med. J. 2009, 338, b481. 19213768 10.1136/bmj.b481 PMC2643441

[166] F. Hayashi , H. Takimoto , K. Yoshita , N. Yoshiike , Br. J. Nutr. 2006, 96, 1154. 17181892 10.1017/bjn20061921

[167] A. Soubry , S. K. Murphy , F. Wang , Z. Huang , A. C. Vidal , B. F. Fuemmeler , J. Kurtzberg , A. Murtha , R. L. Jirtle , J. M. Schildkraut , C. Hoyo , Int. J. Obes. 2015, 39, 650. 10.1038/ijo.2013.193 PMC4048324 24158121

[168] A. Soubry , L. Guo , Z. Huang , C. Hoyo , S. Romanus , T. Price , S. K. Murphy , Clin. Epigenetics 2016, 8, 51. 27158277 10.1186/s13148-016-0217-2 PMC4859994

[169] A. Soubry , J. M. Schildkraut , A. Murtha , F. Wang , Z. Huang , A. Bernal , J. Kurtzberg , R. L. Jirtle , S. K. Murphy , C. Hoyo , BMC Med. 2013, 11, 29. 23388414 10.1186/1741-7015-11-29 PMC3584733

[170] C. Gallou‐Kabani , A. Gabory , J. Tost , M. Karimi , S. Mayeur , J. Lesage , E. Boudadi , M. S. Gross , J. Taurelle , A. Vigé , C. Breton , PLoS One 2010, 5, p. e14398. 21200436 10.1371/journal.pone.0014398 PMC3006175

[171] J. St‐Pierre , M. F. Hivert , P. Perron , P. Poirier , S. P. Guay , D. Brisson , L. Bouchard , Epigenetics 2012, 7, 1125. 22907587 10.4161/epi.21855 PMC3469454

[172] R. C. Laker , T. S. Lillard , M. Okutsu , M. Zhang , K. L. Hoehn , J. J. Connelly , Z. Yan , Diabetes 2014, 63, 1605. 24430439 10.2337/db13-1614 PMC5860829

[173] H. Masuyama , Y. Hiramatsu , Endocrinology 2012, 153, 2823. 22434078 10.1210/en.2011-2161

[174] T. Fullston , E. M. C. O. Teague , N. O. Palmer , M. J. DeBlasio , M. Mitchell , M. Corbett , J. A. Owens , M. Lane , FASEB J. 2013, 27, 4226. 23845863 10.1096/fj.12-224048

[175] G. Zhao‐Jia , L. Shi‐Ming , F. Lin , L. Qiu‐Xia , L. Huang , W. Yan‐Chang , Y. Hou , Z. M. Han , H. Schatten , S. Qing‐Yuan , Environ. Health Perspect. 2014, 122, 159. 24316659 10.1289/ehp.1307047 PMC3915265

[176] J. Zhang , F. Zhang , X. Didelot , K. D. Bruce , F. R. Cagampang , M. Vatish , M. Hanson , H. Lehnert , A. Ceriello , C. D. Byrne , BMC Genomics 2009, 10, 478. 19835573 10.1186/1471-2164-10-478 PMC2770530

[177] K. J. Claycombe , E. O. Uthus , J. N. Roemmich , L. K. Johnson , W. T. Johnson , J. Nutr. 2013, 143, 1533. 23946348 10.3945/jn.113.178038

[178] R. S. Strakovsky , X. Zhang , D. Zhou , Y. X. Pan , J. Physiol. 2011, 589, 2707. 21486814 10.1113/jphysiol.2010.203950 PMC3112549

[179] F. Gaccioli , V. White , E. Capobianco , T. L. Powell , A. Jawerbaum , T. Jansson , Biol. Reprod. 2013, 89, 96. 24006279 10.1095/biolreprod.113.109702

[180] E. Rother , R. Kuschewski , M. A. A. Alcazar , A. Oberthuer , I. Bae‐Gartz , C. Vohlen , B. Roth , J. Dötsch , Endocrinology 2011, 153, 770. 22147015 10.1210/en.2011-1589

[181] Y. Ding , J. Li , S. Liu , L. Zhang , H. Xiao , H. Chen , R. B. Petersen , K. Huang , L. Zheng , Int. J. Obes. 2014, 38, 198. 10.1038/ijo.2013.98 23736364

[182] A. Marco , T. Kisliouk , T. Tabachnik , N. Meiri , A. Weller , FASEB J. 2014, 28, 4148. 24928196 10.1096/fj.14-255620 PMC5395737

[183] K. F. Yang , W. Cai , J. L. Xu , W. Shi , J. Mol. Endocrinol. 2012, 49, 107. 22696509 10.1530/JME-12-0046

[184] M. J. Morris , H. Chen , Int. J. Obes. 2009, 33, 115. 10.1038/ijo.2008.213 18982008

[185] X. Zhang , R. Strakovsky , D. Zhou , Y. Zhang , Y. X. Pan , J. Nutr. 2011, 141, 1254. 21562236 10.3945/jn.111.139576

[186] J. Li , J. Huang , J. S. Li , H. Chen , K. Huang , L. Zheng , J. Hepatol. 2012, 56, 900. 22173165 10.1016/j.jhep.2011.10.018

[187] G. Q. Chang , V. Gaysinskaya , O. Karatayev , S. F. Leibowitz , J. Neurosci. 2008, 28, 12107. 19005075 10.1523/JNEUROSCI.2642-08.2008 PMC2752048

[188] S. F. Ng , R. C. Lin , D. R. Laybutt , R. Barres , J. A. Owens , M. J. Morris , Nature 2010, 467, 963. 20962845 10.1038/nature09491

[189] K. D. Bruce , F. R. Cagampang , M. Argenton , J. Zhang , P. L. Ethirajan , G. C. Burdge , A. C. Bateman , G. F. Clough , L. Poston , M. A. Hanson , J. M. McConnell , Hepatology 2009, 50, 1796. 19816994 10.1002/hep.23205

[190] M. Suter , P. Bocock , L. Showalter , M. Hu , C. Shope , R. McKnight , K. Grove , R. Lane , K. Aagaard‐Tillery , FASEB J. 2011, 25, 714. 21097519 10.1096/fj.10-172080 PMC3228348

[191] M. A. Suter , A. Chen , M. S. Burdine , M. Choudhury , R. A. Harris , R. H. Lane , J. E. Friedman , K. L. Grove , A. J. Tackett , K. M. Aagaard , FASEB J. 2012, 26, 5106. 22982377 10.1096/fj.12-212878 PMC3509051

[192] N. G. Ashino , K. N Saito , F. D. Souza , F. S. Nakutz , E. A. Roman , L. A. Velloso , A. S. Torsoni , M. A. Torsoni , J. Nutr. Biochem. 2012, 23, 341. 21543214 10.1016/j.jnutbio.2010.12.011

[193] B. E. Grayson , P. R. Levasseur , S. M. Williams , M. S. Smith , D. L. Marks , K. L. Grove , Endocrinology 2010, 151, 1622. 20176722 10.1210/en.2009-1019 PMC2850229

[194] K. J. Dudley , D. M. Sloboda , K. L. Connor , J. Beltrand , M. H. Vickers , PloS One 2011, 6, e21662. 21779332 10.1371/journal.pone.0021662 PMC3133558

[195] Z. Vucetic , J. Kimmel , T. M. Reyes , Neuropsychopharmacology 2011, 36, 1199. 21326195 10.1038/npp.2011.4 PMC3077442

[196] N. Murabayashi , T. Sugiyama , L. Zhang , Y. Kamimoto , T. Umekawa , N. Ma , N. Sagawa , Eur. J. Obstet. Gynecol. Reprod. Biol. 2013, 169, 39. 23453296 10.1016/j.ejogrb.2013.02.003

[197] J. Bentham , A. C. Michell , H. Lockstone , D. Andrew , J. E. Schneider , N. A. Brown , S. Bhattacharya , Hum. Mol. Genet. 2010, 19, 3394. 20566713 10.1093/hmg/ddq251 PMC2916708

[198] R. O. Benatti , A. M. Melo , F. O. Borges , L. M. Ignacio‐Souza , L A. P. Simino , M. Milanski , L. A. Velloso , M. A. Torsoni , A. S. Torsoni , Br. J. Nutr. 2014, 111, 2112. 24666709 10.1017/S0007114514000579

[199] S. J. Borengasser , P. Kang , J. Faske , H. Gomez‐Acevedo , M. L. Blackburn , T. M. Badger , K. Shankar , PloS One 2014, 9, e84209. 24416203 10.1371/journal.pone.0084209 PMC3886966

[200] M. A. Suter , J. Ma , P. M. Vuguin , K. Hartil , A. Fiallo , R. A. Harris , M. J. Charron , K. M. Aagaard , Am. J. Obstet. Gynecol. 2014, 210, 463‐e1. 24793723 10.1016/j.ajog.2014.01.045 PMC4368445

[201] R. E. W. Kavey , J. Am. Diet. Assoc. 2010, 110, 1456. 20869483 10.1016/j.jada.2010.07.028

[202] F. B. Hu , V. S. Malik , Physiol. Behav. 2010, 100, 47. 20138901 10.1016/j.physbeh.2010.01.036 PMC2862460

[203] E. L. Sullivan , M. S. Smith , K. L. Grove , Neuroendocrinology 2010, 93, 1. 21079387 10.1159/000322038 PMC3700139

[204] I. Y. Khan , V. Dekou , G. Douglas , R. Jensen , M. A. Hanson , L. Poston , P. D. Taylor , Am. J. Physiol. Regulat. Integrat. Comparat. Physiol. 2005, 288, R127. 10.1152/ajpregu.00354.2004 15308487

[205] B. Sun , R. H. Purcell , C. E. Terrillion , J. Yan , T. H. Moran , K. L. Tamashiro , Diabetes 2012, 61, 2833. 22751689 10.2337/db11-0957 PMC3478561

[206] K. M. Godfrey , A. Sheppard , P. D. Gluckman , K. A. Lillycrop , G. C. Burdge , C. McLean , J. Rodford , J. L. Slater‐Jefferies , E. Garratt , S. R. Crozier , B. S. Emerald , Diabetes 2011, 60, 1528. 21471513 10.2337/db10-0979 PMC3115550

[207] C. L. Relton , A. Groom , B. S. Pourcain , A. E. Sayers , D. C. Swan , N. D. Embleton , M. S. Pearce , S. M. Ring , K. Northstone , J. H. Tobias , J. Trakalo , PloS One 2012, 7, e31821. 22431966 10.1371/journal.pone.0031821 PMC3303769

[208] E. Perkins , S. K. Murphy , A. P. Murtha , J. Schildkraut , R. L. Jirtle , W. Demark‐Wahnefried , M. R. Forman , J. Kurtzberg , F. Overcash , Z. Huang , C. Hoyo , J. Pediatr. 2012, 161, 31. 22341586 10.1016/j.jpeds.2012.01.015 PMC3360130

[209] P. Kuehnen , M. Mischke , S. Wiegand , C. Sers , B. Horsthemke , S. Lau , T. Keil , Y. A. Lee , A. Grueters , H. Krude , PLoS Genet. 2012, 8, e1002543. 22438814 10.1371/journal.pgen.1002543 PMC3305357

[210] W. Perng , M. Mora‐Plazas , C. Marín , L. S. Rozek , A. Baylin , E. Villamor , PloS One 2013, 8, e62587. 23638120 10.1371/journal.pone.0062587 PMC3640064

[211] A. Groom , C. Potter , D. C. Swan , G. Fatemifar , D. M. Evans , S. M. Ring , V. Turcot , M. S. Pearce , N. D. Embleton , G. D. Smith , J. C. Mathers , Diabetes 2012, 61, 391. 22190649 10.2337/db11-1039 PMC3266428

[212] R. Waterland , Nestle Nutr. Workshop Ser. Pediatr. Program. 2005, 56, 157. 10.1159/000086298 16632951

[213] D. Graf , R. Di Cagno , F. Fåk , H. J. Flint , M. Nyman , M. Saarela , B. Watzl , Microb. Ecol. Health Dis. 2015, 26, 26164. 25656825 10.3402/mehd.v26.26164 PMC4318938

[214] A. Brevik , S. E. Vollset , G. S. Tell , H. Refsum , P. M. Ueland , E. B. Loeken , C. A. Drevon , L. F. Andersen , Am. J. Clin. Nutr. 2005, 81, 434. 15699232 10.1093/ajcn.81.2.434

[215] L. M. Bermejo , A. Aparicio , P. Andrés , A. M. López‐Sobaler , R. M. Ortega , Public Health Nutr. 2007, 10, 266. 17288624 10.1017/S1368980007246580

[216] R. W. Welch , P. C. Mitchell , Br. Med. Bull. 2000, 56, 1. 10885101 10.1258/0007142001902923

[217] J. L. Slavin , D. Jacobs , L. Marquart , Cr. Rev. Food Sci. Nutr. 2000, 40, 309. 10.1080/10408690091189176 10943592

[218] M. C. Nicoli , M. Anese , M. Parpinel , Trends Food Sci. Technol. 1999, 10, 94.

[219] S. Stender , J. Dyerberg , Ann. Nutr. Metab. 2004, 48, 61. 14679314 10.1159/000075591

[220] M. U. Imam , M. Ismail , D. J. Ooi , N. H. Azmi , N. Sarega , K. W. Chan , M. I. Bhanger , Cr. Rev. Biotechnol. 2016, 36, 585. 10.3109/07388551.2014.995586 25641328

[221] F. B. Hu , J. E. Manson , W. C. Willett , J. Am. Coll. Nutr. 2001, 20, 5. 11293467 10.1080/07315724.2001.10719008

[222] M. U. Imam , M. Ismail , in Genomics, Proteomics and Metabolomics in Nutraceuticals and Functional Foods, 2nd ed. (Eds: Bagchi D., Swaroop A., Bagchi M.), Wiley‐Blackwell, Hoboken, NJ, USA 2015, p. 504.

[223] Q. Sun , D. Spiegelman , R. M. van Dam , M. D. Holmes , V. S. Malik , W. C. Willett , F. B. Hu , Arch. Intern. Med. 2010, 170, 961. 20548009 10.1001/archinternmed.2010.109 PMC3024208

[224] A. Nanri , T. Mizoue , M. Noda , Y. Takahashi , M. Kato , M. Inoue , S. Tsugane , Am. J. Clin. Nutr. 2010, 92, 1468. 20980490 10.3945/ajcn.2010.29512

[225] E. A. Hu , A. Pan , V. Malik , Q. Sun , Br. Med. J. 2012, 344, e1454. 22422870 10.1136/bmj.e1454 PMC3307808

[226] M. U. Imam , M. Ismail , D. J. Ooi , N. Sarega , A. Ishaka , Mol. Nutr. Food Res. 2015, 59, 180. 25329877 10.1002/mnfr.201400396

[227] M. A. Hullar , B. C. Fu , Cancer J. 2014, 20, 170. 24855003 10.1097/PPO.0000000000000053 PMC4267719

[228] M. W. Bourassa , I. Alim , S. J. Bultman , R. R. Ratan , Neurosci. Lett. 2016, 625, 56. 26868600 10.1016/j.neulet.2016.02.009 PMC4903954

[229] T. M. S. Wolever , J. ‐L. Chiasson , Br. J. Nutr. 2000, 84, 57–61. 10961161

[230] E. C. Nelissen , A. P. van Montfoort , J. C. Dumoulin , J. L. Evers , Hum. Reprod. Update 2011, 17, 397. 20959349 10.1093/humupd/dmq052

[231] M. A. Maccani , C. J. Marsit , Am. J. Reprod. Immunol. 2009, 62, 78. 19614624 10.1111/j.1600-0897.2009.00716.x PMC2813777

[232] B. Paul , S. Barnes , W. Demark‐Wahnefried , C. Morrow , C. Salvador , C. Skibola , T. O. Tollefsbol , Clin. Epigenet. 2015, 7, 112. 10.1186/s13148-015-0144-7 PMC4609101 26478753

[233] T. Rönn , P. Volkov , C. Davegårdh , T. Dayeh , E. Hall , A. H. Olsson , E. Nilsson , A. Tornberg , M. D. Nitert , K. F. Eriksson , H. A. Jones , PLoS Genet. 2013, 9, e1003572. 23825961 10.1371/journal.pgen.1003572 PMC3694844

[234] J. Denham , B. J. O'Brien , J. T. Harvey , F. J. Charchar , Epigenomics 2015, 7, 717. 25864559 10.2217/epi.15.29

[235] J. Ntanasis‐Stathopoulos , J. G. Tzanninis , A. Philippou , M. Koutsilieris , J. Musculoskeletal Neuronal Interact. 2013, 13, 133. 23728100

[236] Y. Nomura , L. Lambertini , A. Rialdi , M. Lee , E. Y. Mystal , M. Grabie , I. Manaster , N. Huynh , J. Finik , M. Davey , K. Davey , Reprod. Sci. 2014, 21, 131. 23765376 10.1177/1933719113492206 PMC3857768

[237] F. Guénard , Y. Deshaies , K. Cianflone , J. G. Kral , P. Marceau , M. C. Vohl , Proc. Nat. Acad. Sci. USA 2013, 110, 11439. 23716672 10.1073/pnas.1216959110 PMC3710842

[238] S. J. Van Dijk , P. L. Molloy , H. Varinli , J. L. Morrison , B. S. Muhlhausler , members of EpiSCOPE , Int. J. Obes. 2015, 39, 85. 10.1038/ijo.2014.34 24566855

[239] D. Berglind , P. Müller , M. Willmer , I. Sinha , P. Tynelius , E. Näslund , K. Dahlman‐Wright , F. Rasmussen , Obesity 2016, 24, 250. 26637991 10.1002/oby.21340

[240] J. Smith , K. Cianflone , S. Biron , F. S. Hould , S. Lebel , S. Marceau , O. Lescelleur , L. Biertho , S. Simard , J. G. Kral , P. Marceau , J. Clin. Endocrinol. Metab. 2009, 94, 4275. 19820018 10.1210/jc.2009-0709

[241] F. Guénard , A. Tchernof , Y. Deshaies , K. Cianflone , J. G. Kral , P. Marceau , M. C. Vohl , J. Obes. 2013, 2013, 492170. 23840945 10.1155/2013/492170 PMC3693160

[242] G. C. Sharp , D. A. Lawlor , R. C. Richmond , A. Fraser , A. Simpkin , M. Suderman , H. A. Shihab , O. Lyttleton , W. McArdle , S. M. Ring , T. R. Gaunt , G. Davey Smith , C. L. Relton , Int. J. Epidemiol. 2015, 44, 1288. 25855720 10.1093/ije/dyv042 PMC4588865

[243] S. B. Eaton , S. B. Eaton , Comp. Biochem. Physiol., Part A: Mol. Integr. Physiol. 2003, 136, 153. 10.1016/s1095-6433(03)00208-3 14527637

[244] J. P. Rey‐Lopez , G. Vicente‐Rodríguez , M. Biosca , L. A. Moreno , Nutr. Metab. Cardiovasc. Dis. 2008, 18, 242. 18083016 10.1016/j.numecd.2007.07.008

[245] E. G. Wilmot , C. L. Edwardson , F. A. Achana , M. J. Davies , T. Gorely , L. J. Gray , K. Khunti , T. Yates , S. J. Biddle , Diabetologia 2012, 55, 2895. 22890825 10.1007/s00125-012-2677-z

[246] J. H. O'Keefe , R. Vogel , C. J. Lavie , L. Cordain , Prog. Cardiovasc. Dis. 2011, 53, 471. 21545934 10.1016/j.pcad.2011.03.009

[247] J. H. O'Keefe , R. Vogel , C. J. Lavie , L. Cordain , Am. J Med. 2010, 123, 1082. 20843503 10.1016/j.amjmed.2010.04.026

[248] US Department of Health and Human Services , The health consequences of smoking: a report of the Surgeon General, https://www.surgeongeneral.gov/library/reports/50‐years‐of‐progress/full‐report.pdf (accessed April 2017).

[249] M. Öberg , M. S. Jaakkola , A. Woodward , A. Peruga , A. Prüss‐Ustün , Lancet 2011, 377, 139. 21112082 10.1016/S0140-6736(10)61388-8

[250] V. S. Knopik , M. A. Maccani , S. Francazio , J. E. McGeary , Dev. Psychopathol. 2012, 24, 1377. 23062304 10.1017/S0954579412000776 PMC3581096

[251] W. Besingi , Å. Johansson , Hum. Mol. Genet. 2014, 23, 2290. 24334605 10.1093/hmg/ddt621

[252] R. Joehanes , A. C. Just , R. E. Marioni , L. C. Pilling , L. M. Reynolds , P. R. Mandaviya , W. Guan , T. Xu , C. E. Elks , S. Aslibekyan , H. Moreno‐Macias , J. A. Smith , J. A. Brody , R. Dhingra , P. Yousefi , J. S. Pankow , S. Kunze , S. H. Shah , A. F. McRae , K. Lohman , J. Sha , D. M. Absher , L. Ferrucci , W. Zhao , E. W. Demerath , J. Bressler , M. L. Grove , T. Huan , C. Liu , M. M. Mendelson , C. Yao , D. P. Kiel , A. Peters , R. Wang‐Sattler , P. M. Visscher , N. R. Wray , J. M. Starr , J. Ding , C. J. Rodriguez , N. J. Wareham , M. R. Irvin , D. Zhi , M. Barrdahl , P. Vineis , S. Ambatipudi , A. G. Uitterlinden , A. Hofman , J. Schwartz , E. Colicino , L. Hou , P. S. Vokonas , D. G. Hernandez , A. B. Singleton , S. Bandinelli , S. T. Turner , E. B. Ware , A. K. Smith , T. Klengel , E. B. Binder , B. M. Psaty , K. D. Taylor , S. A. Gharib , B. R. Swenson , L. Liang , D. L. DeMeo , G. T. O'Connor , Z. Herceg , K. J. Ressler , K. N. Conneely , N. Sotoodehnia , S. L. Kardia , D. Melzer , A. A. Baccarelli , J. B. van Meurs , I. Romieu , D. K. Arnett , K. K. Ong , Y. Liu , M. Waldenberger , I. J. Deary , M. Fornage , D. Levy , S. J. London , Circ Cardiovasc Genet. 2016, 9, 436. 27651444 10.1161/CIRCGENETICS.116.001506 PMC5267325

[253] C. A. Markunas , Z. Xu , S. Harlid , P. A. Wade , R. T. Lie , J. A. Taylor , A. J. Wilcox , Environ. Health Perspect. 2014, 122, 1147. 24906187 10.1289/ehp.1307892 PMC4181928

[254] M. Suter , J. Ma , A. Harris , L. Patterson , K. A. Brown , C. Shope , L. Showalter , A. Abramovici , K. M. Aagaard‐Tillery , Epigenetics 2011, 6, 1284. 21937876 10.4161/epi.6.11.17819 PMC3242811

[255] M. Suter , A. Abramovici , L. Showalter , M. Hu , C. D. Shope , M. Varner , K. Aagaard‐Tillery , Metabolism 2010, 59,1481. 20462615 10.1016/j.metabol.2010.01.013 PMC2921565

[256] M. A. Maccani , M. Avissar‐Whiting , C. E. Banister , B. McGonnigal , J. F. Padbury , C. J. Marsit , Epigenetics 2010, 5, 583. 20647767 10.4161/epi.5.7.12762 PMC2974801

[257] S. K. Murphy , A. Adigun , Z. Huang , F. Overcash , F. Wang , R. L. Jirtle , J. M. Schildkraut , A. P. Murtha , E. S. Iversen , C. Hoyo , Gene 2012, 494, 36. 22202639 10.1016/j.gene.2011.11.062 PMC3627389

[258] V. K. Rehan , J. Liu , E. Naeem , J. Tian , R. Sakurai , K. Kwong , O. Akbari , J. S. Torday , BMC Med. 2012, 10, 129. 23106849 10.1186/1741-7015-10-129 PMC3568737

[259] C. V. Breton , H. M. Byun , M. Wenten , F. Pan , A. Yang , F. D. Gilliland , Am. J. Respir. Crit. Care Med. 2009, 180, 462. 19498054 10.1164/rccm.200901-0135OC PMC2742762

[260] B. R. Joubert , S. E. Håberg , D. A. Bell , R. M. Nilsen , S. E. Vollset , Ø. Midttun , P. M. Ueland , M. C. Wu , W. Nystad , S. D. Peddada , S. J. London , Cancer Epidemiol. Biomarkers Prev. 2014, 23, 1007. 24740201 10.1158/1055-9965.EPI-13-1256 PMC4140220

[261] L. K. Küpers , X. Xu , S. A. Jankipersadsing , A. Vaez , S. la Bastide‐van Gemert , S. Scholtens , I. M. Nolte , R. C. Richmond , C. L. Relton , J. F. Felix , L. Duijts , Int. J. Epidemiol. 2015, 44, 1224. 25862628 10.1093/ije/dyv048 PMC4588868

[262] C. Nevin , M. Carroll , J. Hum. Genet. Clin. Embryol. 2015, 1, 004.

[263] M. Vuolo , J. Staff , Pediatrics 2013, 132, e568. 23918887 10.1542/peds.2013-0067 PMC3876755

[264] M. Ungerer , J. Knezovich , M. Ramsay , Alcohol Res. Curr. Rev. 2013, 35, 37. 10.35946/arcr.v35.1.05 PMC3860424 24313163

[265] M. L. Masemola , L. van der Merwe , Z. Lombard , D. Viljoen , M. Ramsay , Front. Genet. 2015, 6, 85. 25806045 10.3389/fgene.2015.00085 PMC4354390

[266] B. Y. Lee , S. Y. Park , H. M. Ryu , C. Y. Shin , K. N. Ko , J. Y. Han , G. Koren , Y. H. Cho , Alcohol. Clin. Exp. Res. 2015, 39, 239. 25656446 10.1111/acer.12635

[267] B. I. Laufer , J. Kapalanga , C. A. Castellani , E. J. Diehl , L. Yan , S. M. Singh , Epigenomics 2015, 7, 1259. 26178076 10.2217/epi.15.60

[268] C. P. Chen , P. Kuhn , J. P. Advis , D. K. Sarkar , J. Neurochem. 2006, 97, 1026. 16686691 10.1111/j.1471-4159.2006.03839.x

[269] D. Govorko , R. A. Bekdash , C. Zhang , D. K. Sarkar , Biol. Psychiatry 2012, 72, 378. 22622000 10.1016/j.biopsych.2012.04.006 PMC3414692

[270] Y. F. Ngai , D. C. Sulistyoningrum , R. O'Neill , S. M. Innis , J. Weinberg , A. M. Devlin , Am. J. Physiol. Regulat. Integrat. Comparat. Physiol. 2015, 309, R613. 10.1152/ajpregu.00075.2015 PMC4591382 26180184

[271] O. Gangisetty , O. Wynne , S. Jabbar , C. Nasello , D. K. Sarkar , PloS One 2015, 10, e0140699. 26509893 10.1371/journal.pone.0140699 PMC4624904

[272] C. R. Zhang , M. F. Ho , M. C. S. Vega , T. H. Burne , S. Chong , Epigenet. Chromatin 2015, 8, 40. 10.1186/s13072-015-0032-6 PMC4587775 26421062

[273] D. K. Sarkar , Addict. Biol. 2016, 21, 23. 25581210 10.1111/adb.12186 PMC7250160

[274] World Health Organization , Global status report on alcohol and health 2014, http://apps.who.int/iris/bitstream/10665/112736/1/9789240692763_eng.pdf (accessed: April 2017).

[275] A. W. Musk , N. H. De Klerk , Respirology 2003, 8, 286. 14528877 10.1046/j.1440-1843.2003.00483.x

[276] M. A. Schuckit , T. L. Smith , J. Stud. Alcohol 1997, 58, 141. 9065891 10.15288/jsa.1997.58.141

[277] World Health Organization , Air pollution estimates and health impact, http://www.who.int/mediacentre/news/releases/2016/air‐pollution‐estimates/en/ (accessed: March 2017).

[278] M. K. Skinner , M. Manikkam , R. Tracey , C. Guerrero‐Bosagna , M. Haque , E. E. Nilsson , BMC Med. 2013, 11, 228. 24228800 10.1186/1741-7015-11-228 PMC3853586

[279] D. Valvi , M. A. Mendez , D. Martinez , J. O. Grimalt , M. Torrent , J. Sunyer , M. Vrijheid , Environ. Health Perspect. 2012, 120, 451. 22027556 10.1289/ehp.1103862 PMC3295349

[280] M. Manikkam , R. Tracey , C. Guerrero‐Bosagna , M. K. Skinner , PloS One 2013, 8, e55387. 23359474 10.1371/journal.pone.0055387 PMC3554682

[281] J. Hong , F. Chen , X. Wang , Y. Bai , R. Zhou , Y. Li , L. Chen , Mol. Cell. Endocrinol. 2016, 427, 101. 26975478 10.1016/j.mce.2016.03.009

[282] A. Ziv‐Gal , W. Wang , C. Zhou , J. A. Flaws , Toxicol. Appl. Pharmacol. 2015, 284, 354. 25771130 10.1016/j.taap.2015.03.003 PMC4410077

[283] G. Li , H. Chang , W. Xia , Z. Mao , Y. Li , S. Xu , Toxicol. Lett. 2014, 228, 192. 24793715 10.1016/j.toxlet.2014.04.012

[284] M. Miao , X. Zhou , Y. Li , O. Zhang , Z. Zhou , T. Li , W. Yuan , R. Li , D. K. Li , Andrology 2014, 2, 138. 24293158 10.1111/j.2047-2927.2013.00166.x

[285] M. Lombó , C. Fernández‐Díez , S. González‐Rojo , C. Navarro , V. Robles , M. P. Herráez , Environ. Pollut. 2015, 206, 667. 26322593 10.1016/j.envpol.2015.08.016

[286] M. D. Anway , C. Leathers , M. K. Skinner , Endocrinology 2006, 147, 5515. 16973726 10.1210/en.2006-0640 PMC5940332

[287] M. Manikkam , R. Tracey , C. Guerrero‐Bosagna , M. K. Skinner , PloS One 2012, 7, e46249. 23049995 10.1371/journal.pone.0046249 PMC3458876

[288] M. Manikkam , R. Tracey , C. Guerrero‐Bosagna , M. K. Skinner , Reprod. Toxicol. 2012, 34, 708. 22975477 10.1016/j.reprotox.2012.08.010 PMC3513590

[289] R. Chamorro‐García , M. Sahu , R. J. Abbey , J. Laude , N. Pham , B. Blumberg , Environ. Health Perspect. 2013, 121, 359. 23322813 10.1289/ehp.1205701 PMC3621201

[290] M. Manikkam , M. M. Haque , C. Guerrero‐Bosagna , E. E. Nilsson , M. K. Skinner , PloS One 2014, 9, e102091. 25057798 10.1371/journal.pone.0102091 PMC4109920

[291] A. Debost‐Legrand , C. Warembourg , C. Massart , C. Chevrier , N. Bonvallot , C. Monfort , F. Rouget , F. Bonnet , S. Cordier , Environ. Res. 2016, 146, 207. 26775002 10.1016/j.envres.2016.01.005

[292] R. Tracey , M. Manikkam , C. Guerrero‐Bosagna , M. K. Skinner , Reprod. Toxicol. 2013, 36, 104. 23453003 10.1016/j.reprotox.2012.11.011 PMC3587983

[293] D. MacArthur , Nat. Biotechnol. 2016, 34, 512. 27065009 10.1038/nbt.3555

[294] E. Barrett‐Connor , K. Khaw , Circulation 1984, 69, 1065. 6713610 10.1161/01.cir.69.6.1065

[295] M. T. Scheuner , S. J. Wang , L. J. Raffel , S. K. Larabell , J. I. Rotter , Am. J. Med. Genet. 1997, 71, 315. 9268102 10.1002/(sici)1096-8628(19970822)71:3<315::aid-ajmg12>3.0.co;2-n

[296] F. J. Rauscher , Cancer Res. 2005, 65, 11229. 16357122 10.1158/0008-5472.CAN-65-24-ED1

[297] M. Esteller , Carcinogenesis 2006, 27, 1121. 16699174 10.1093/carcin/bgl033

[298] Human Epigenome Consortium , Human Epigenome Project, http://www.epigenome.org/index.php?page=project (accessed: July 2017).

